# Efficacy and Safety of Chemotherapy Regimens in Advanced or Metastatic Bladder and Urothelial Carcinomas: An Updated Network Meta-Analysis

**DOI:** 10.3389/fphar.2019.01507

**Published:** 2020-01-15

**Authors:** Hong-Chen Qu, Yan Huang, Zhong-Yi Mu, Hang Lv, Qing-Peng Xie, Kai Wang, Bin Hu

**Affiliations:** Department of Urological Surgery, Cancer Hospital of China Medical University/Liaoning Cancer Hospital & Institute, Shenyang, China

**Keywords:** metastatic, advanced, urothelial carcinoma, bladder cancer, treatment, meta-analysis

## Abstract

**Background:** Gemcitabine plus cisplatin (GC) and methotrexate, vinblastine, adriamycin, and cisplatin (MVAC) have been the first-line treatments for advanced or metastatic urothelial carcinoma (AMUC). However, their effects are unsatisfactory, and more drugs and regimens still need to be explored.

**Objective:** We aimed to comprehensively compare all possible regimens with GC or MVAC in randomized controlled trials (RCTs) by network meta-analysis.

**Methods:** We searched the PubMed, Embase, and Cochrane databases for RCTs that evaluated regimens compared to GC or MVAC on AMUC patients. The major outcomes were progression-free survival (PFS), overall survival (OS), and objective response rate (ORR). A network meta-analysis was used to assess the effectiveness and safety of the included treatment regimens, and the regimens were then clustered by the average linkage method.

**Results:** A total of 19 trials that assessed 3,363 AMUC patients were included. For PFS, paclitaxel plus GC (PGC) was significantly superior to GC (log hazard ratio (HR): −0.16; 95% confidence interval (CI): −0.32, 0.00) with a moderate level of reliability. However, there was no significant difference between PGC and MVAC (log HR: −0.03; 95% CI: −0.27, 0.20). For OS, PGC was significantly superior to GC (log HR:−0.17; 95% CI: −0.33, −0.00) with a moderate reliability level but not significantly different from MVAC (log HR: −0.10; 95% CI: −0.35, 0.15). Analysis of ORR showed that PGC was superior to MVAC (log odds ratio (OR): 0.59; 95% CI: 0.02, 1.16) with a low reliability level and GC (log OR: 0.41; 95% CI: 0.12, 0.71) with a moderate reliability level. In the cluster results, PGC and sorafenib plus GC (GCS) exhibited relative advantages in efficiency, followed by MVAC and apatorsen plus GC (GCA); however, PGC, gemcitabine plus carboplatin (GP), and MVAC had more serious side effects.

**Conclusions:** In our analysis, PGC was superior to MVAC and GC in only the ORR results and superior to GC in the OS and PFS results but was not significantly different from MVAC. More individualized therapies with targeted drugs need to be studied.

## Introduction

Urothelial carcinoma is the most common type of bladder cancer and can also occur in other parts of the urinary system, such as the renal pelvis, ureter, and urethra (Bianchi et al., 2014; Venyo, 2014). The stages of UC include nonmuscle-invasive urothelial carcinoma (NMIUC), which has a high recurrence and progression rate (30–65%) (Jallad et al., 2014) and can develop into muscle-invasive urothelial carcinoma (MIUC) (Resnick et al., 2013) and advanced or metastatic urothelial carcinoma (AMUC) (Necchi et al., 2017).

NMIUC of the bladder is usually treated by instillation. Bacillus Calmette-Guerin is more effective than other chemotherapy methods (Boehm et al., 2017; Huang et al., 2017) and can be combined with epirubicin (EPI) and mitomycin C (MMC) to prevent recurrence (Zhuo et al., 2016; Wu et al., 2017). Radical cystectomy with bilateral pelvic lymphadenectomy is usually used to prevent recurrence in MIUC patients and is usually supplemented with the chemotherapeutic strategy of methotrexate, vinblastine, adriamycin, and cisplatin (MVAC) (Kim et al., 2016).

For AMUC, gemcitabine plus cisplatin (GC) or MVAC is generally used as a first-line chemotherapy regimen (Ismaili et al., 2011; Racioppi et al., 2012). However, their effects are unsatisfactory, and more drugs and regimens still need to be explored. Taxanes, vinflunine, and immunotherapy are often used as second-line treatments (Narayanan et al., 2015; Holmsten et al., 2016; Bellmunt et al., 2017). In 2016, the FDA rapid review approved atezolizumab as a treatment for AMUC, but subsequent studies confirmed that the drug did not meet the original survival target (Ning et al., 2017).

There are many meta-analysis studies on chemotherapy strategies for AMUC. The combination of multiple chemotherapy drugs (Giannatempo et al., 2016; Raggi et al., 2016; Wu et al., 2016; Necchi et al., 2017) and immune checkpoint inhibitors is believed to have survival benefits in patients (Li and Wang, 2018; Di Nunno et al., 2018). However, there is still a lack of direct and indirect comparisons among various chemotherapeutic strategies. Therefore, their clinical application is still unclear.

A Bayesian network meta-analysis assessed the safety and efficacy of various therapeutic strategies for AMUC patients with advanced urothelial cancer who underwent chemotherapy and showed that paclitaxel or sorafenib combined with GC had better survival advantages but serious side effects. However, bladder site tumors were neglected in this study, and the results of the direct and indirect comparisons between the chemotherapeutic strategies were also not clearly listed (Wang et al., 2018). In our study, we updated the above work and used the frequentist method to analyze randomized controlled trials (RCTs) comparing MVAC or GC with other strategies to provide guidance for the clinical treatment of AMUC.

## Methods

This network meta-analysis was pereformed according to the Preferred Reporting Items for Systematic Reviews Network Meta-Analyses (PRISMA-NMA) guidelines (Hutton et al., 2015).

## Search Strategy

We searched online databases including PubMed, Embase, and Cochrane Library up to 10 April 2019. Search terms included the following: “bladder,” “intravesical,” “urothelial,” “urethral,” “urothelium,” “urinary,” “neoplasms,” “cancer,” “malignant,” “carcinoma,” “tumor,” “advanced,” “metastases,” “metastatic,” “late-stage,” “random*,” “cisplatin,” “platin,” “carboplatin,” “gemcitabine,” “methotrexate,” “vinblastine,” and “doxorubicin” (**Supplementary Table 1**). The references of the relevant reviews were also checked to ensure that no additional relevant studies were inadvertently omitted. Only published English-language trials were considered.

## Data Selection

Studies were eligible if the following criteria were met: 1: included AMUC patients; 2: used RCTs; 3: had one arm with either GC, GP, or MVAC treatment; and 4: examined either progression-free survival (PFS), overall survival (OS), or objective response rate (ORR). PFS and OS could be in the form of a hazard ratio (HR) from a Cox regression model or predictable survival curve. The exclusion criteria included the following: 1: studies that did not include AMUC patients or did not report AMUC subset results; 2: non-RCTs; 3: RCTs that did not have a GC or MVAC arm or those that compared GC or MVAC with a placebo or blank control; 4: studies that compared the same drugs but different application strategies; and 5: studies that did not obtain any PFS, OS, or ORR results. Conference summaries, commentaries, and editorials were also excluded.

## Data Extraction

The extracted contents included the first author name, publication year, type of patients, sample size, age, experimental intervention, control intervention, and follow-up period. The major outcomes were PFS, OS, and ORR. The secondary outcome was severe adverse events (SAEs) that had a grade> = 3 according to the National Cancer Institute (NCI) Common Terminology Criteria for Adverse Events. We assessed the methodological quality of the included trials using a risk of bias approach according to the methods described by the Cochrane Collaboration, which include seven specified domains (Higgins et al., 2011). In addition, we also applied Grading of Recommendation Assessment, Development and Evaluation (GRADE) guidance to evaluate the quality of the network analysis results with four levels graded from high (best) to very low (worst) (Yan and Xu, 2018). This method takes into account the design level of direct and indirect comparisons, the inconsistency of direct and indirect comparison results, the imprecision of results, and the large effect results that can improve the level of evidence.

## Statistical Analysis

The PFS and OS results are represented by HRs and their 95% confidence intervals (CIs). For studies with survival curve results, the results were gathered from the curve. The ORRs and adverse events (AEs) are represented as binary data. The odds ratios (ORs) and their 95% CIs were calculated by extracting the frequencies of events. We used a frequentist framework random-effects model for mixed multiple treatment comparisons (Greco et al., 2015). Global and local inconsistencies between direct and indirect sources of evidence were assessed by the fit of consistency and inconsistency models and the difference between direct and indirect estimates in all closed loops, respectively. To rank the treatments for each outcome, we used sureface under the cumulative ranking (SUCRA) probabilities (Li et al., 2015). After obtaining SUCRA values, the major outcomes and SAEs were clustered by the average linkage clustering method. This exploratory clustering method avoids the intereference of individual deviation samples on the overall results. Comparison-adjusted funnel plots were used to determine small-study effects in the analysis (Trinquart et al., 2012). Data analyses were pereformed using STATA software (version 14.0; STATA Corporation, College Station, TX, USA).

## Results

### Literature Search

**Figure 1:**
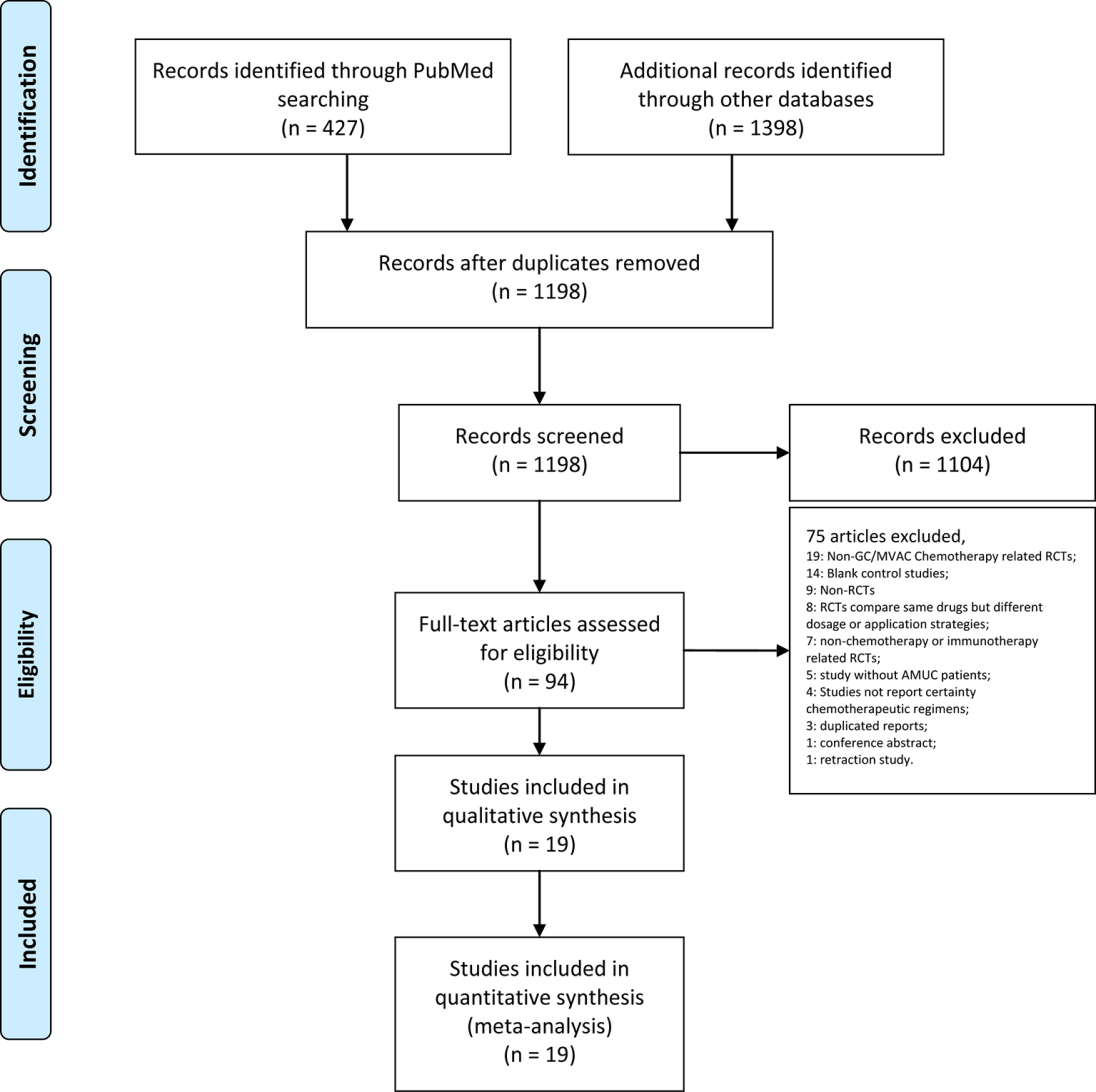
PRISMA flow chart illustrating the selection process of the studies included in our analysis.

After the database searches, 427 articles were obtained from PubMed, 891 articles from Embase and 446 trials and 61 reviews from the Cochrane Library. After removing duplications, 1,198 articles remained. Then, 1,104 articles were excluded after screening the titles and abstracts. The full texts of the remaining 94 articles were assessed. Studies were further excluded due to the following reasons: non-GC/MVAC chemotherapy-related RCTs (Wu et al., 2016); blank control studies (Narayanan et al., 2015); non-RCTs (Zhuo et al., 2016); RCTs comparing the same drugs but different dosage or application strategies (Wu et al., 2017); nonchemotherapy- or immunotherapy-related RCTs (Huang et al., 2017); studies without AMUC patients (Necchi et al., 2017); studies that did not report certain chemotherapeutic regimens (Resnick et al., 2013); duplicated reports (Jallad et al., 2014); conference abstract (Bianchi et al., 2014); and retraction study (Bianchi et al., 2014). Finally, 19 articles were included in our analysis (Logothetis et al., 1990; Bellmunt et al., 1997; Saxman et al., 1997; McCaffrey et al., 1997; Siefker-Radtke et al., 2002; Dreicer et al., 2004; Bamias et al., 2004; von der Maase et al., 2005; Lorusso et al., 2005; Dogliotti et al., 2007; Bellmunt et al., 2012; De Santis et al., 2012; Sternberg et al., 2013; Bamias et al., 2013; Hussain et al., 2014; Krege et al., 2014; Miller et al., 2016; Bellmunt et al., 2017; Cao et al., 2018) (**Figure 1**, **Table 1**).

**Table 1:** Characteristics of the included studies.

Author	Year	Location	Sample size	Age #	Type of patients	Intervention	Abbr.	Control	Abbr.	Outcomes	Follow-up ##
Cao Y (Cao et al., 2018)	2018	China	53	60 (41–75)	Advanced or metastatic bladder cancer	Gemcitabine;cisplatin	GC	Pemetrexed;cisplatin	PC	PFS;OS;ORR;SAE	Open
Bellmunt J (Bellmunt et al., 2017)	2017	Multicenter	183	64 (34–84)	Advanced urothelial cancer	Gemcitabin;cisplatin;apatorsen	GCA	Gemcitabine;cisplatin	GC	PFS;OS;ORR;SAE	Open
Hussain M (Hussain et al., 2014)	2014	US	88	60.9 (32.8-79.9)	Advanced urothelial carcinoma	Gemcitabine;cisplatin	GC	Gemcitabine;cisplatin;cetuximab	GCCET	PFS;OS;ORR;SAE	2-5 Y
Miller K (Miller et al., 2016)	2014	Multicenter	105	66 (41–84)	Advanced or metastatic transitional cell carcinoma of the urothelium	Gemcitabin;cisplatin;Gefitinib	GCG	Gemcitabine;cisplatin	GC	PFS;OS;ORR;SAE	Open
Sternberg CN (Sternberg et al., 2013)	2013	Multicenter	337	64 (35–85)	Locally advanced/metastatic urothelial tract or bladder cancer	Larotaxel;cisplatin	LC	Gemcitabine;cisplatin	GC	PFS;OS;ORR;SAE	Open
Krege S (Krege et al., 2014)	2013	multicenter	89	67.3 (NA)	Locally advanced and/or metastatic urothelial cancer	Gemcitabine;cisplatin;sorafenib	GCS	Gemcitabine;cisplatin;placebo	GC	PFS;OS;ORR;SAE	>18 M
Bamias A (Bamias et al., 2013)	2012	Greece	126	65. 5 (34–80)	Metastatic or relapsed urothelial cancer	Methotrexate;vinblastine;doxorubicin;cisplatin	MVAC	Gemcitabine;cisplatin	GC	PFS;OS;ORR;SAE	52.1 (0.1-82.5) M
Bellmunt J (Bellmunt et al., 2012)	2012	multicenter	626	61 (27–80)	Locally advanced and/or metastatic urothelial cancer	Paclitaxel;gemcitabine;cisplatin	PGC	Gemcitabine;cisplatin	GC	PFS;OS;ORR;SAE	4.6 (0-6.8) Y
De Santis M (De Santis et al., 2012)	2012	Austria	238	71 (34–87)	Advanced urothelial cancer	Gemcitabine;carboplatin	GP	Methotrexate;carboplatin;vinblastine	MCAVI	PFS;OS;ORR;SAE	4.5 (0-7.8) Y
Dogliotti L (Dogliotti et al., 2007)	2007	Italy and Turkey	110	67 (32–80)	Advanced transitional cell carcinoma of the urothelium	Gemcitabine;cisplatin	GC	Gemcitabine;carboplatin	GP	PFS;OS;ORR;SAE	Open
von der Maase H (von der Maase et al., 2005)	2005	Multicenter	405	63 (NA)	Locally advanced or metastatic transitional cell carcinoma of the urothelium	Gemcitabine;cisplatin	GC	methotrexate;vinblastine;doxorubicin;cisplatin	MVAC	PFS;OS;ORR;SAE	Open
Lorusso V (Lorusso et al., 2005)	2004	Italy	85	68 (48–76)	Advanced transitional cell carcinoma of the urothelium	Paclitaxel;gemcitabine;cisplatin	PGC	Gemcitabine;cisplatin	GC	PFS;OS;ORR;SAE	100 W
Dreicer R (Dreicer et al., 2004)	2004	US	80	64 (NA)	Advanced carcinoma of the urothelium	Methotrexate;vinblastine;doxorubicin;cisplatin	MVAC	Carboplatin;paclitaxel	CP	PFS;OS;ORR;SAE	Open
Bamias A (Bamias et al., 2004)	2004	Multicenter	220	65 (32–75)	Advanced urothelial carcinoma	Docetaxel;cisplatin	DC	Methotrexate;vinblastine;doxorubicin;cisplatin	MVAC	PFS;OS;ORR;SAE	25.3 (3.2-51) M
Siefker-Radtke AO (Siefker-Radtke et al., 2002)	2002	US	172	67 (17–83)	Metastatic or unresectable urothelial cancer	Intereferon α2b;fluorouracil;cisplatin	FAP	Methotrexate;vinblastine;doxorubicin;cisplatin	MVAC	PFS;ORR;SAE	Open
Bellmunt J (Bellmunt et al., 1997)	1997	Spain	47	65 (36–75)	Advanced bladder carcinoma	Methotrexate;carboplatin;vinblastine	MCAVI	Methotrexate;vinblastine;doxorubicin;cisplatin	MVAC	PFS;ORR;SAE	18 (6–60) M
Saxman SB (Saxman et al., 1997)	1997	US	255	66 (30–79)	Metastatic urothelial carcinoma	Methotrexate;vinblastine;doxorubicin;cisplatin	MVAC	Cisplatin	CIS	PFS;OS;ORR;SAE	>6 Y
McCaffrey JA (McCaffrey et al., 1997)	1997	US	34	62 (40–82)	Advanced transitional cell carcinoma	Gallium nitrate;fluorouracil	GF	Methotrexate;vinblastine;doxorubicin;cisplatin	MVAC	OS;ORR;SAE	Open
Logothetis CJ (Logothetis et al., 1990)	1990	US	110	66 (34–78)	Metastatic urothelial tumors	Cisplatin;cyclophosphamide;adriamycin	CCA	Methotrexate;vinblastine;doxorubicin;cisplatin	MVAC	ORR;OS	160 W

NA, not available.

#, Median (Minimum–Maximum); ##, Open: follow-up until death or disease progression; Y, year; M, month; W, week.

**Figure 2:**
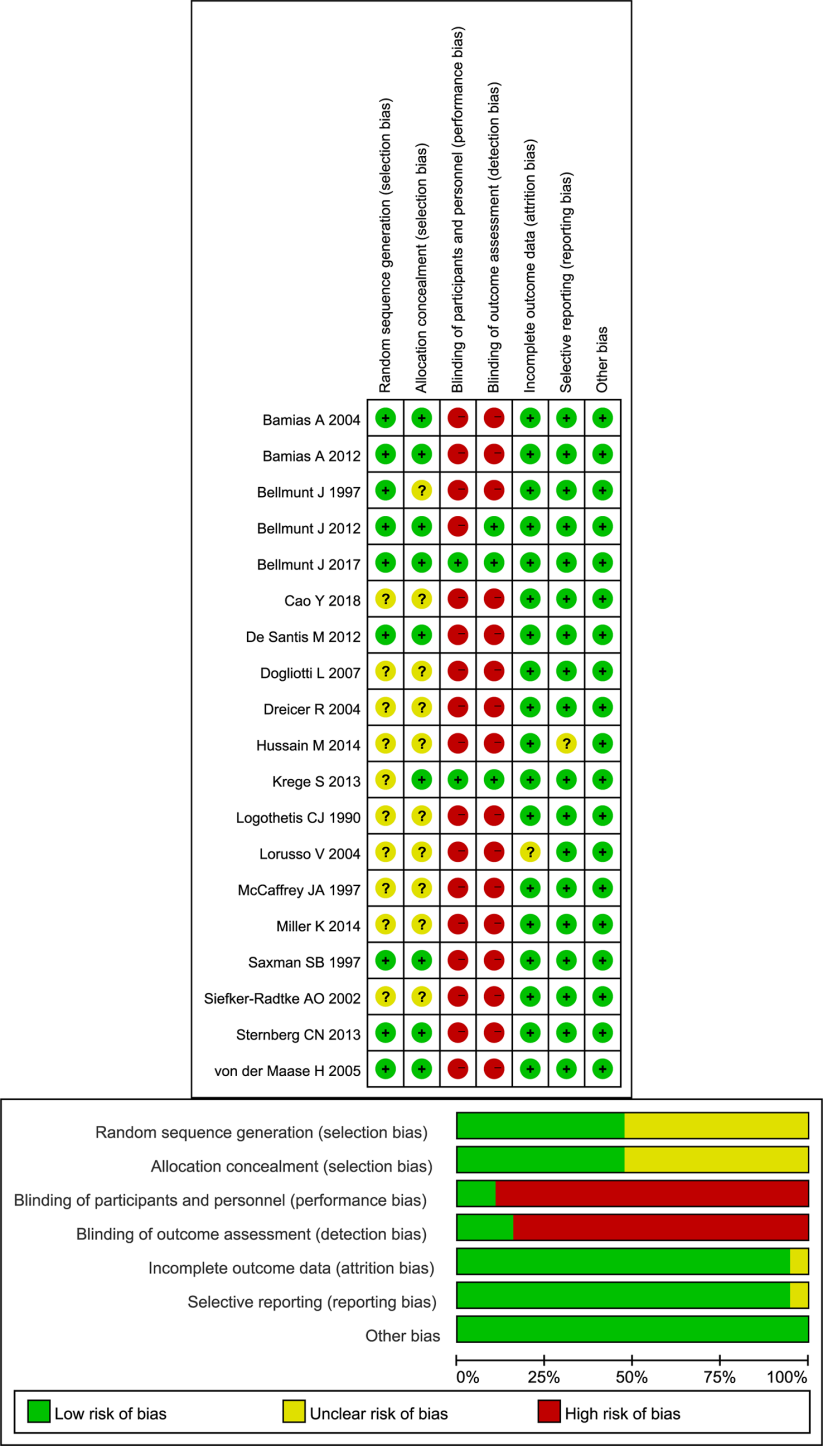
Risk of bias graph of each included study.

The publication time of the included studies had a long time span, ranging from 1990 to 2018. A total of 3363 AMUC patients were enrolled in the study. The median age was 60–70 years old. Three articles included only advanced bladder cancer patients. Two studies reported different outcomes from the same cohort, and we combined the outcomes of these two studies (von der Maase et al., 2000; von der Maase et al., 2005) (**Table 1**). All included studies were of RCT design, but in some studies, the generation of random sequences and random masking were not clearly described. Three studies were blinded (Bellmunt et al., 2012; Krege et al., 2014; Bellmunt et al., 2017). Because the main evaluation results are objective, the quality of the research was generally acceptable (**Figure 2**).

## Results of the Network Meta-Analysis

**Figure 3:**
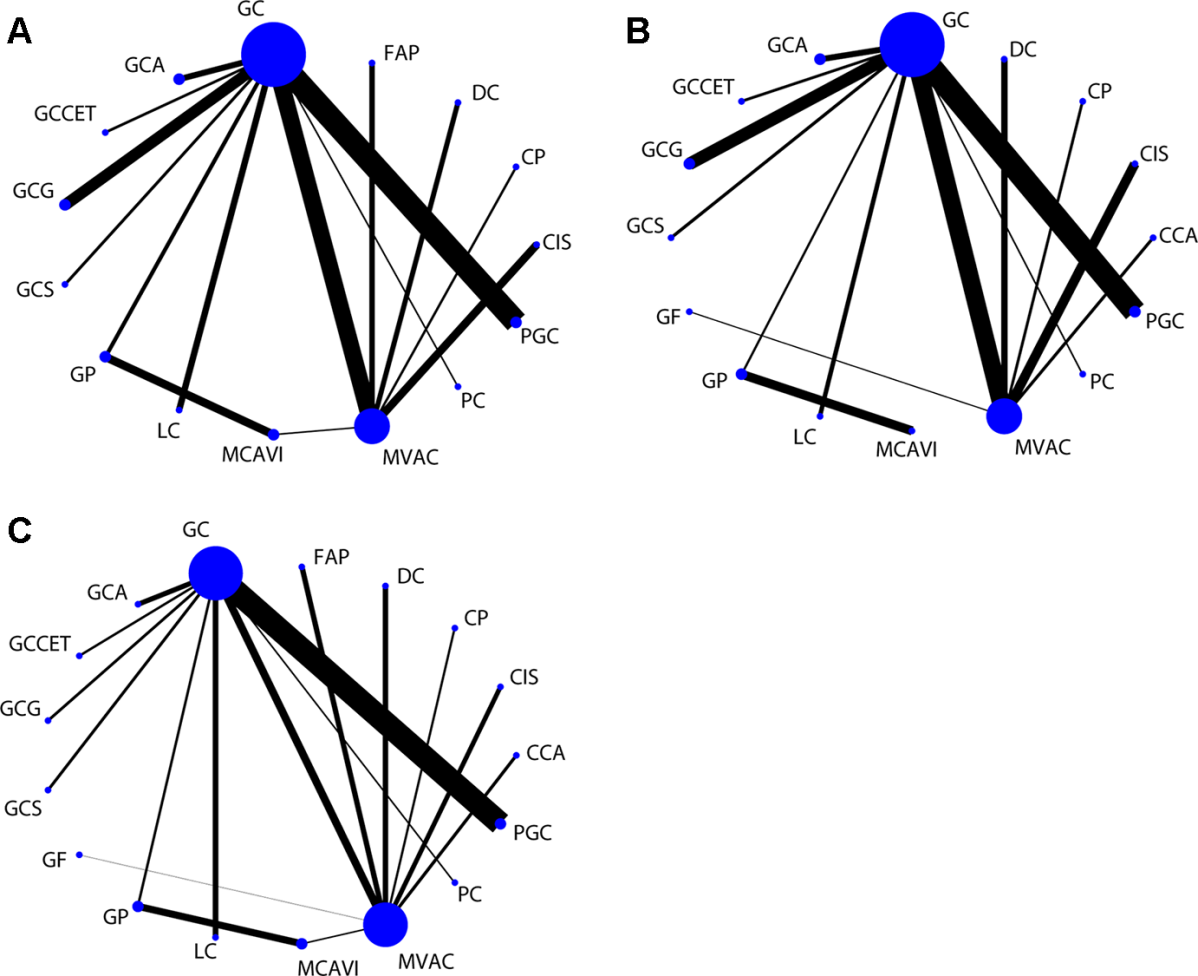
Network of comparisons for the treatment strategies included in the analyses. **(A)** PFS; **(B)** OS; **(C)** ORR. Strategy abbreviations are listed in **Table 1**.

**Figure 4:**
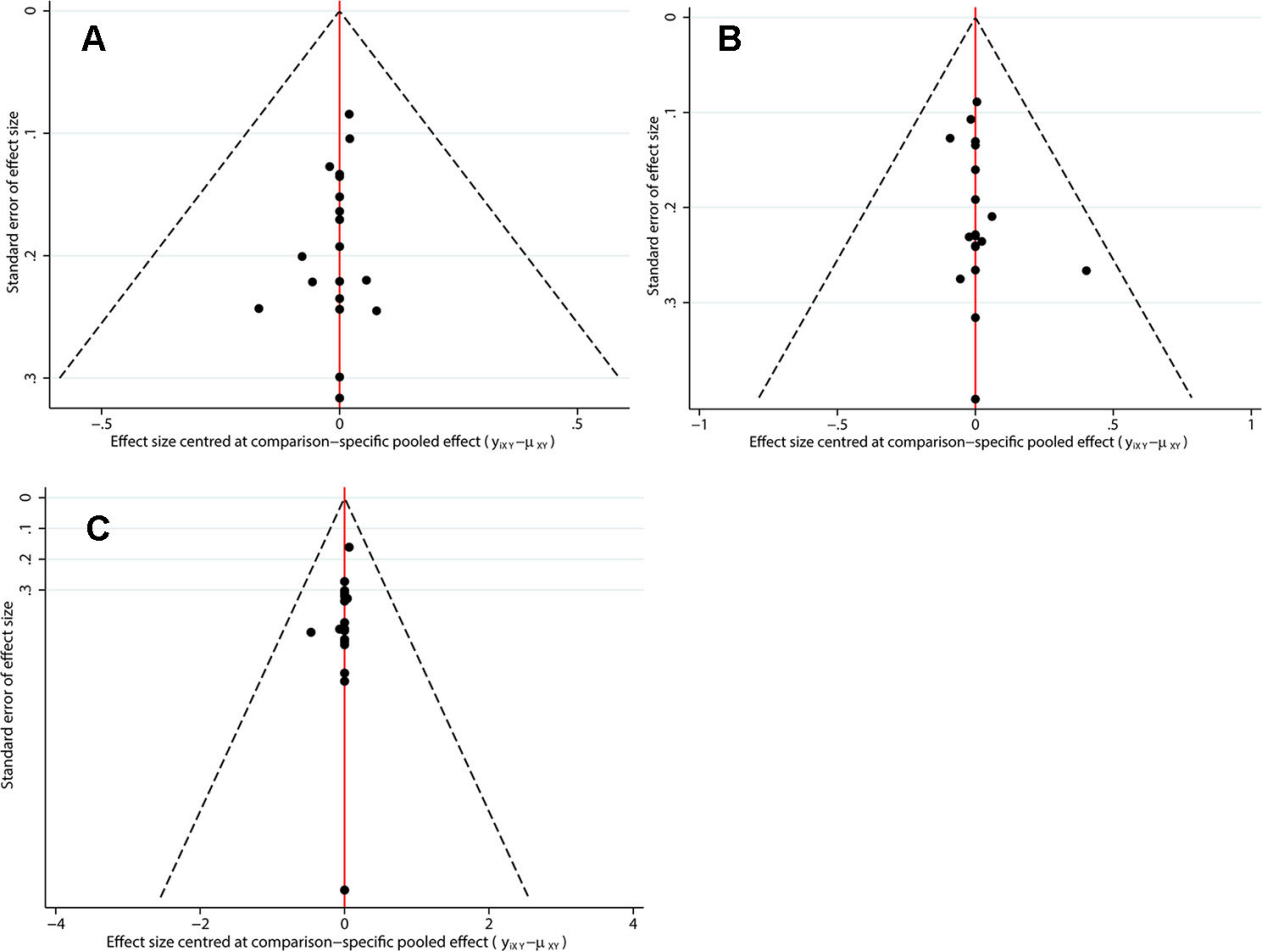
Comparison-adjusted funnel plots for assessing outcomes. **(A)** PFS; **(B)** OS; **(C)** ORR.

In the network meta-analysis of PFS, 15 treatment strategies were analyzed (**Figure 3A**). There were nine GC-related comparisons and 6 MVAC-related comparisons. An inconsistency plot was used to assume loop-specific heterogeneity, and one quadratic loop showed that there was no significant difference between the direct and indirect comparisons (inconsistency factor: 0.33, 95% CI: 01.12) (**Supplementary Figure 1**). Global inconsistency was also not detected in the analysis (p = 0.412). In the network comparisons, paclitaxel plus GC (PGC) was significantly superior to GC (log HR: −0.16; 95% CI: −0.32, 0.00) with a moderate level of reliability. However, there was no significant difference between PGC and MVAC (log HR: −0.03; 95% CI: −0.27, 0.20). MVAC was superior to methotrexate, carboplatin, and vinblastine (MCAVI) (log HR: −0.43; 95% CI: −0.82, −0.05), larotaxel plus cisplatin (LC) (log HR: −0.64; 95% CI: −0.98, −0.29), GP (log HR: −0.36; 95% CI: −0.71, 0.00), docetaxel plus cisplatin (DC) (log HR: −0.55; 95% CI: −0.88, −0.21), and cisplatin (log HR: −0.78; 95% CI: −1.05,−0.52). LC was inferior to GC (log HR: 0.51; 95% CI: 0.21, 0.81), and GC was superior to DC (log HR: −0.42; 95% CI: −0.80, −0.05) and cisplatin (log HR: −0.66; 95% CI:−0.98, −0.34) (**Supplementary Table 2**). A comparison-adjusted funnel plot did not suggest that there was any publication bias (**Figure 4A**).

For the OS results, sixteen treatment regimens were analyzed (**Figure 3B**). Inconsistency analysis did not detect global inconsistency (p = 0.646), and no loops were found in the network comparisons. In the network comparisons, cisplatin, cyclophosphamide plus adriamycin (CCA) was inferior to MVAC (log HR: 0.80; 95% CI: 0.35, 1.25) and GC (log HR: 0.73; 95% CI: 0.25, 1.22) with a low evidence level. PGC was significantly superior to GC (log HR: −0.17; 95% CI: −0.33, 0.00) with a moderate reliability level but not significantly different from MVAC (log HR: −0.10; 95% CI: −0.35, 0.15). MVAC was superior to MCAVI (log HR: −1.41; 95% CI: −2.02, −0.79) and GP (log HR: −1.47; 95% CI: −2.02, −0.91) with a low reliability level and DC (log HR: −0.42; 95% CI: −0.73, −0.10) and cisplatin (log HR: −0.51; 95% CI: −0.77, −0.26) with a moderate reliability level. MCAVI (log HR: 1.34; 95% CI: 0.75, 1.92) and GP (log HR: 1.40; 95% CI: 0.88, 1.92) were both inferior to GC with low reliability levels. GC was superior to cisplatin (log HR: −0.44; 95% CI: −0.76, −0.12) with moderate reliability levels (**Supplementary Table 3**). There was no publication bias detected in the analysis (**Figure 4B**).

Analysis of the ORR results included nine GC-related comparisons and eight MVAC-related comparisons (**Figure 3C**). The inconsistency analysis showed that no global (p = 0.8229) or local inconsistencies (inconsistency factor: 0.19; 95% CI: 0.00, 1.84) were detected (**Supplementary Figure 2**). In the network comparisons, CCA was inferior to GC (log OR: −0.96; 95% CI: −1.89, −0.02). PGC was superior to MVAC (log OR: 0.59; 95% CI: 0.02, 1.16) with a low reliability level and GC (log OR: 0.41; 95% CI: 0.12, 0.71) with a moderate level. MVAC was superior to gallium nitrate plus fluorouracil (GF) (log OR: 4.79; 95% CI: 2.29, 7.29), intereferon α2b, fluorouracil plus cisplatin (FAP) (log OR: 0.69; 95% CI: 0.08, 1.30), DC (log OR: 0.69; 95% CI: 0.08, 1.29), and cisplatin (log OR: 1.50 95% CI: 0.85, 2.16) with a low reliability level. GC was also superior to GF (log OR: 4.96; 95% CI: 2.42, 7.51), FAP (log OR: 0.87; 95% CI: 0.09, 1.65), DC (log OR: 0.86 95% CI: 0.09, 1.64), and cisplatin (log OR: 1.68; 95% CI: 0.86, 2.50) at low reliability levels (**Supplementary Table 4**). No publication bias was detected (**Figure 4C**).

**Figure 5:**
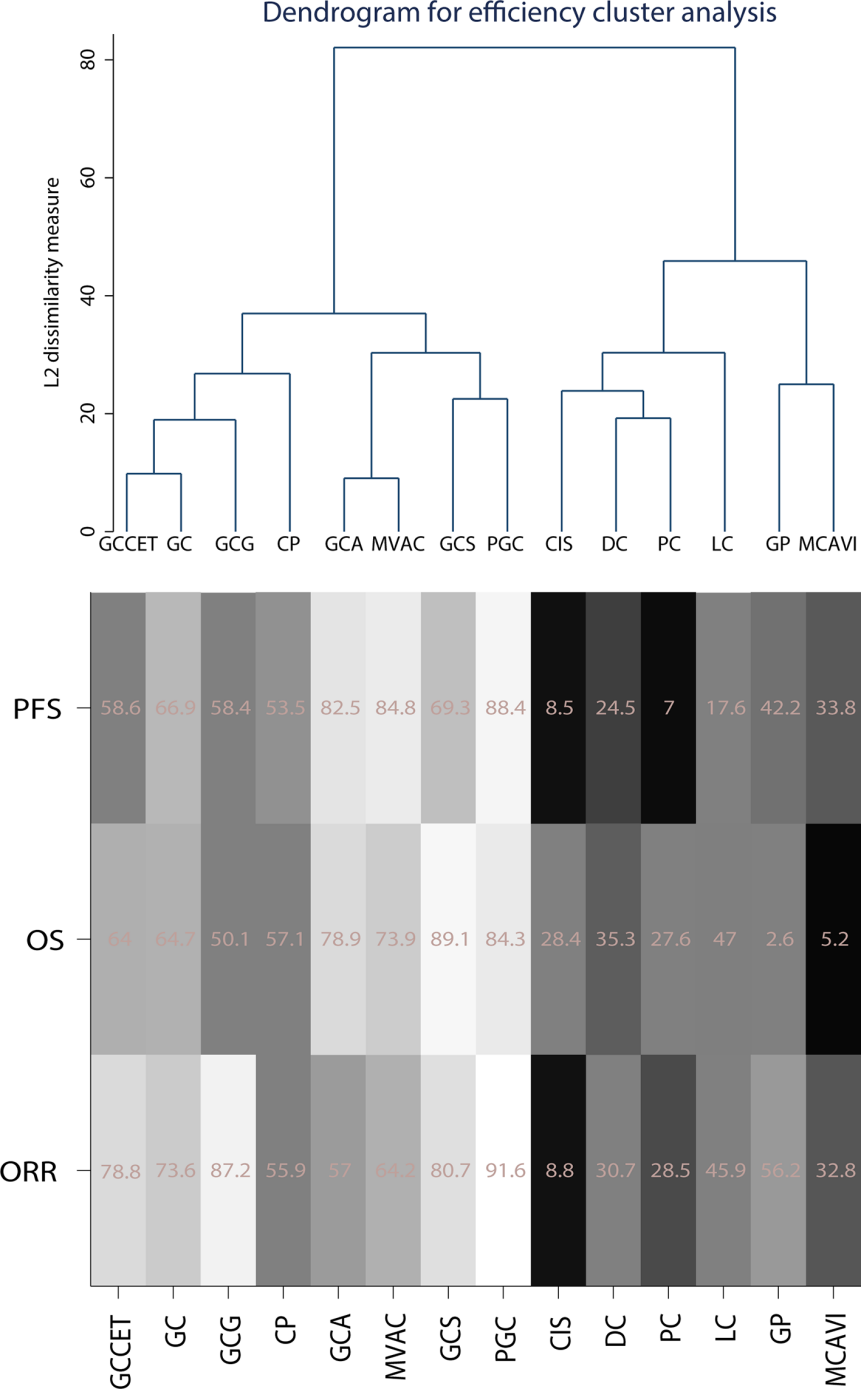
Cluster of efficiency outcomes for the included chemotherapy strategies. Strategy abbreviations are listed in **Table 1**. The SUCRA scores are weighted, with white indicating high scores and black indicating low scores.

The cluster method was generally used to classify and analyze the validity and security of the results in the network analysis. We used the average linkage clustering method to classify the treatment regimens by clustering the efficiency and safety outcomes separately. Fourteen treatment regimens were included in the efficiency cluster analysis, while others were excluded due to a lack of SUCRA results. PGC and GCS had relative advantages in efficiency, followed by MVAC and GCA. In contrast, cisplatin, DC, PC, LC, GP, and MCAVI were considered relatively inefficient (**Figure 5**).

**Figure 6:**
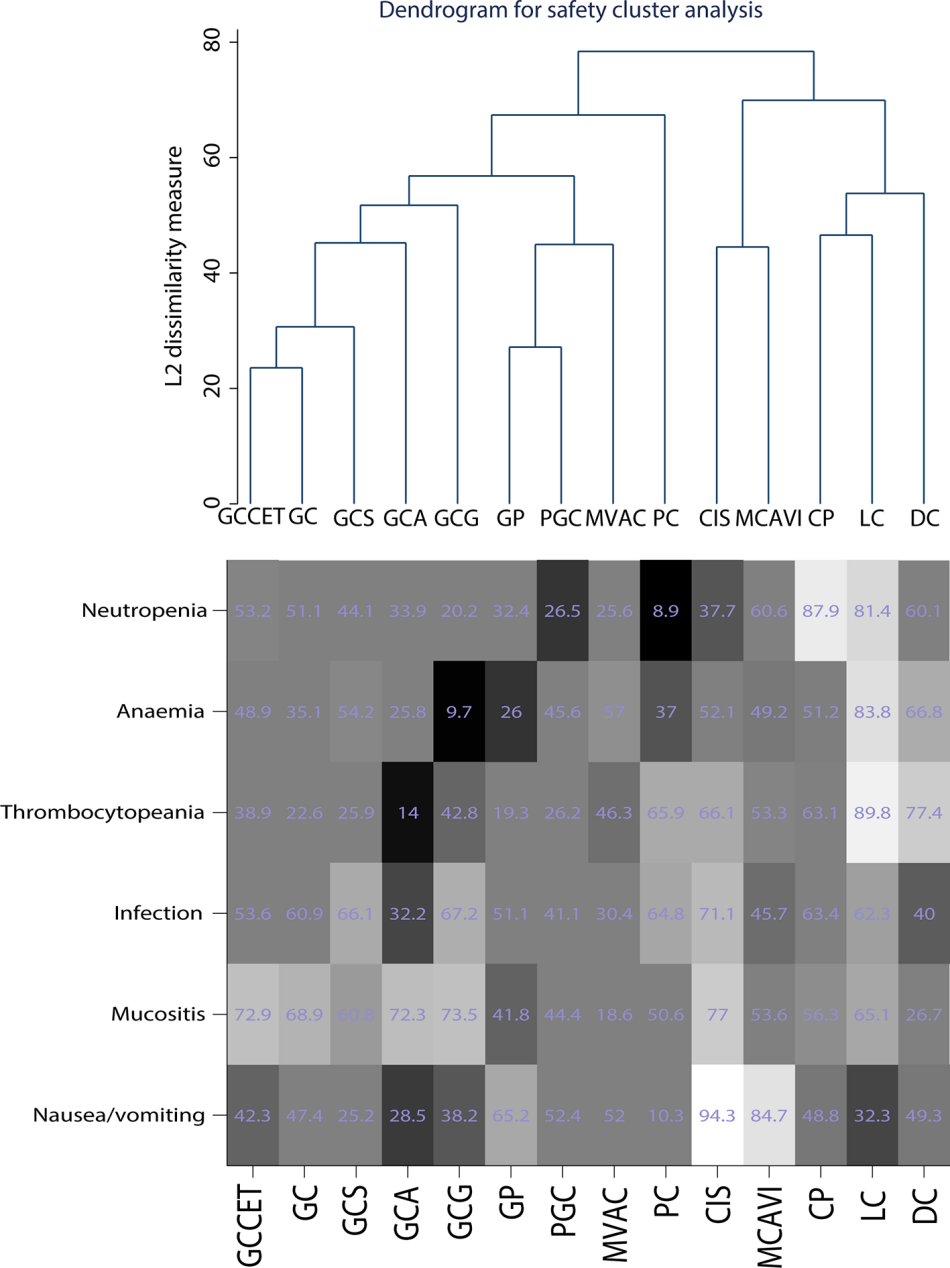
Cluster of safety outcomes for the included chemotherapy strategies.

The cluster results of safety outcomes and SAE (grade> = 3) included analyses of neutropenia, anemia, thrombocytopenia, infection, mucositis, and nausea/vomiting, which are frequently reported. The cluster analysis showed that regimens such as cisplatin, MCAVI, CP, LC, and DC had fewer SAEs but were also less effective. The cytotoxic drugs that have a weak effect on cancer cells may also have a weaker effect on normal cells. GC-related treatment strategies, such as GC, GCCET, GCS, GCA, and gemcitabine, cisplatin, and gefitinib (GCG), had similar SAE clusters. However, PGC, GP, and MVAC exhibited more serious side effects. In general, PGC and MVAC had better therapeutic effects than other regimens but had more serious adverse effects as well. GCS and GCA exhibited a similar efficacy to MVAC and had relatively mild SAEs, similar to GC (**Figure 6**).

## Discussion

There is still a need to explore more effective methods for the treatment of AMUC. Because GC and MVAC are widely used and regarded as effective treatments for AMUC, this study comprehensively analyzed all possible regimens compared to GC or MVAC in RCTs by network meta-analysis. In addition, carboplatin and cisplatin are both platinum-based treatments; therefore, we included GP and GC in the analysis. This work aimed to find more advantageous regimens to guide clinical applications.

In this study, seventeen AMUC treatment regimens were included, and fourteen of them were clustered. No suitable clustering scheme was identified when all results were considered at the same level. Therefore, we applied the average linkage method to merge multiple primary and secondary results. Using this approach, analysis of the efficiency revealed that PGC, GCS, MVAC, and GCA were more effective than CIS, DC, PC, LC, GP, and MCAVI. Analysis of the safety outcomes indicated that GC-related treatment strategies, such as GC, GCCET, GCS, GCA, and GCG, had similar side effects whereas PGC, GP, and MVAC had more serious SAEs.

The exclusion of several studies from this work needs to be explained. Vinflunine-related and lapatinib-related RCTs were excluded because both studies conducted a more than four-week drug-eluting phase after chemotherapy (Garcia-Donas et al., 2017; Powles et al., 2017). Due to the eluting phase, the research drug and chemotherapy were not considered to be a combination treatment strategy. Pembrolizumab was demonstrated to have longer OS rates than the investigator’s choice of chemotherapy for advanced UC patients in a phase 3 trial (Bellmunt et al., 2017). That study was excluded because the chemotherapy regimens were unclear. A granulocyte stimulating factor (GSF)-related RCT comparing GSF-MVAC and MVAC was excluded because GSF is commonly used in AMUC patients with hematologic AEs after chemotherapy (Logothetis et al., 1995). In addition, because GC and MVAC regimens have been proven to be effective for AMUC, blank control studies were not included in the analysis.

Compared with the previous network meta-analysis, the main difference in this study is that advanced bladder cancer patients were included; therefore, more studies and intervention regimens were also included (Wang et al., 2018). A retracted study (Roberts et al., 2006) and a nonrandomized study (Hsieh et al., 2016) were excluded, which were included in the previous analysis. Furthermore, a trastuzumab-related RCT was excluded because of an unclear chemotherapy strategy in the intervention group (Oudard et al., 2015). In addition, this work used the frequentist method instead of the Bayesian method for the network meta-analysis, which further provides the results of direct and indirect comparisons among the included regimens and then classifies the reliability of the results according to the GRADE method. Notably, PGC ranked well in SUCRA scores and was significantly better than MVAC and GC regimens in ORR. However, for OS and PFS, PGC was only significantly better than GC and not significantly different from MVAC.

Although there was no significant survival benefit compared to MVAC, PGC is still a potential chemotherapy regimen for AMUC. In this regimen, cisplatin intereferes with DNA replication and mitosis by crosslinking with DNA. Gemcitabine is a derivative of a cytosine nucleoside that stops the synthesis of DNA strands and results in masked chain termination to avoid normal repair, leading to DNA breakage. These two drugs cooperatively prevent DNA replication and cell separation as a first-line treatment. Compared with GC, PGC had a significantly better ORR, OS, and PFS in this study. Paclitaxel is a microtubule-associated inhibitor that stabilizes microtubule polymers and promotes their assembly to arrest cells in the M phase. However, even the PGC regimen did not provide a significant survival benefit compared to MVAC.

In addition to traditional cytotoxic drugs, targeted drugs have also been researched for AMUC treatment. Sorafenib is a multitarget inhibitor of tyrosine kinase. GCS exhibited similar effects as PGC in the cluster analysis, showing SAEs similar to those observed with GC. However, there was still no significant difference between GCS and GC or MVAC regarding PFS, OS, or ORR. The unsatisfactory therapeutic result of GCS may be due to the small number of patients, or GCS may only be effective for specific AMUC patients. In a recent phase I study of metastatic urothelial carcinoma second-line therapy, sorafenib and vinflunine combined had an OS rate of 7 (1.8–41.7) months and ORR of 41%; however, RCTs are needed to confirm this therapeutic effect (Knievel et al., 2014; Shah et al., 2019). Individualized therapy for these targeted and sensitive drugs may further improve the therapeutic effect. However, in *in vitro* studies, the limited role of sorafenib in the mitogen-activated protein kinase (MAPK) signaling pathway of urothelial cancer cell lines suggests that sorafenib is not very suitable for UC treatment (Knievel et al., 2014).

Gefitinib is an epidermal growth factor receptor tyrosine kinase domain inhibitor. However, in a trial, 13 of 20 metastatic bladder cancer patients had obvious epidermal growth factor receptor (EGFR) expression, and gefitinib did not confer enough survival benefits to patients or improved ORR (Philips et al., 2008). In preclinical studies, gefitinib was demonstrated to reverse the sensitivity of cisplatin and paclitaxel-resistant UC cells, indicating that the potential mechanism of gefitinib requires further research (Wang et al., 2017). As EGFR inhibitors, cetuximab combined with GC also did not achieve the desired effect (Hussain et al., 2014). Lapatinib, a target drug for human epidermal growth factor receptor 2 (HER2), did not significantly improve the therapeutic effect in HER1/HER2-expressing metastatic bladder cancer (MBC) patients. Even in the strong HER1/2 position subgroup, lapatinib did not significantly improve the survival benefit (Powles et al., 2017). In an excluded study, trastuzumab combined with GC or GP was used to treat metastatic urothelial cancer (Oudard et al., 2015). However, the combination did not significantly improve ORR, OS, or PFS in patients. The low incidence of HER2 overexpression in patients suggests that trastuzumab lacks the means of universal application. Therefore, EGFR may not be a desired therapeutic target for AMUC.

Apatorsen, which inhibits the production of heat shock protein 27 (Hsp27), has also been shown to have significant survival benefits for AMUC patients. However, after classification by Hsp27 level, subgrouped patients with <5.7 ng/ml and < = 20.5% exhibited an obvious survival benefit after apatorsen treatment (Rosenberg et al., 2018). The above results suggest that individualized treatment is a research direction for improving treatment effects on AMUC patients.

For other macromolecule-targeted drugs, such as vascular endothelial growth factor inhibitors (e.g., bevacizumab) and PD-L1 inhibitors (e.g., atezolizumab), no related studies were included because none met the inclusion criteria. Although the therapeutic advantage of atezolizumab has been demonstrated in phase II of a single-arm study (Balar et al., 2017), the results of large-scale RCTs are still needed. PD-L1 expression detection was also considered a prediction of the therapeutic effect of atezolizumab (Crist and Balar, 2017). Ultimately, for the application of targeted drugs, the characteristics of AMUC patients, such as target protein expression, need to be a focus of future studies.

## Limitations

There were still several limitations in this work. First, this study included only GC- and MVAC-related RCTs to maintain the continuity of the network analysis and neglected some non-GC- or MVAC-related RCTs. This work did not analyze the differences in drug dosage or application duration among treatment regimens. The small sample sizes of single arms may affect the accuracy and reliability of the results. Finally, as more RCTs are reported, the conclusions may change.

## Author Contributions

Study concepts and design: BH. Literature research: HQ. Data acquisition: YH and KW. Data analysis: ZM and HQ. Statistical analysis: HL and HQ. Manuscript preparation: QX, HQ, and BH. Manuscript editing: HQ and BH. Manuscript review: HQ and BH.

## Conflict of Interest

The authors declare that the research was conducted in the absence of any commercial or financial relationships that could be construed as a potential conflict of interest.

## Supplementary Material

Supplementary Figure 1 Inconsistency plot of loop-specific heterogeneity for PFS.

Supplementary Figure 2 Inconsistency plot of loop-specific heterogeneity for ORR.

Supplementary Table 1 Full electronic search strategy in the PubMed database.

Supplementary Table 2 The PFS results of the chemotherapy strategies according to their relative effect and reliable quality.

Supplementary Table 3 The OS results of the chemotherapy strategies according to their relative effect and reliable quality.

Supplementary Table 4 The ORR results of the chemotherapy strategies according to their relative effect and reliable quality.
