# Supplementary figures and images for: Efficacy and Safety of Chemotherapy Regimens in Advanced or Metastatic Bladder and Urothelial Carcinomas: An Updated Network Meta-Analysis
Source: Front Pharmacol. 2020 Jan 15;10:1507. doi: 10.3389/fphar.2019.01507 (PMC6974923; doi:10.3389/fphar.2019.01507)

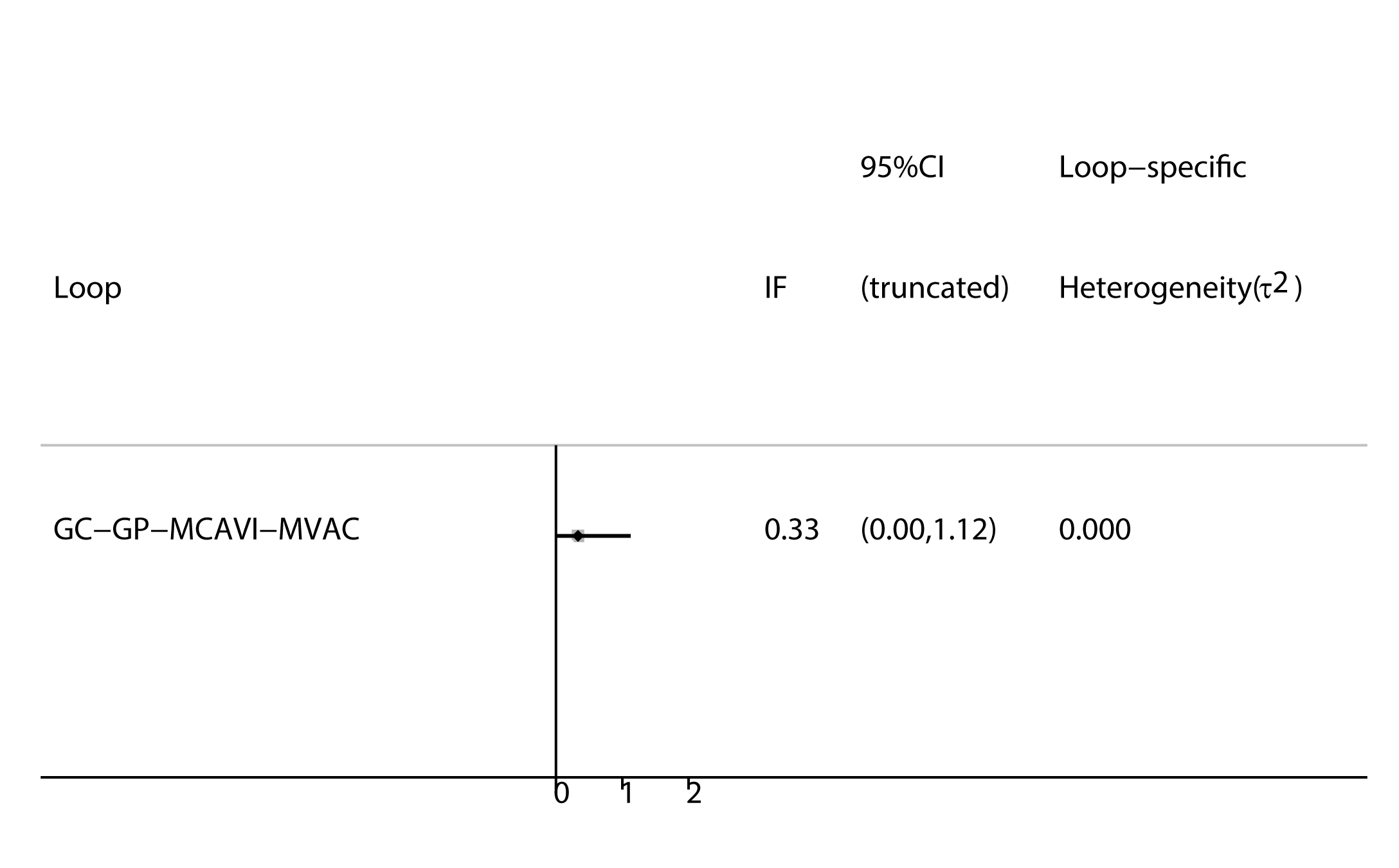

Supplement: Supplementary Figure 1 — Inconsistency plot of loop-specific heterogeneity for PFS. [file Image_1.tif]

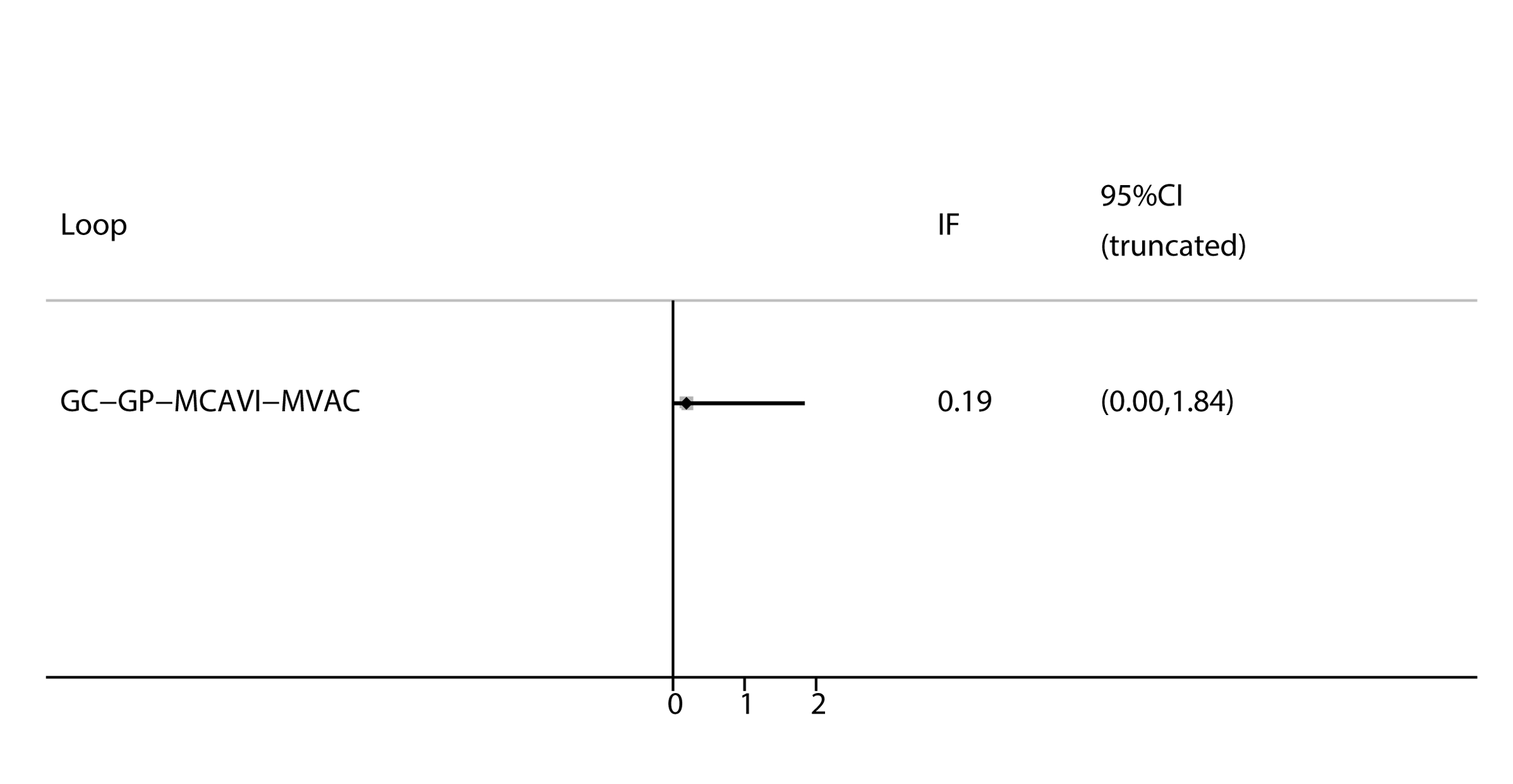

Supplement: Supplementary Figure 2 — Inconsistency plot of loop-specific heterogeneity for ORR. [file Image_2.tif]
