# Supplementary material for: Efficacy and Safety of Chemotherapy Regimens in Advanced or Metastatic Bladder and Urothelial Carcinomas: An Updated Network Meta-Analysis
Source: Front Pharmacol. 2020 Jan 15;10:1507. doi: 10.3389/fphar.2019.01507 (PMC6974923; doi:10.3389/fphar.2019.01507)
Supplement: Supplementary Table 1 — Full electronic search strategy in the PubMed database. [file Table_1.docx]

Supplementary table 1. Full electronic search strategy in PubMed Database.

| Search | Query | Items found |
| --- | --- | --- |
| #6 | Search ((((#1) AND #2) AND #3) AND #4) AND #5 | 427 |
| #5 | Search ((((((cisplatin) OR gemcitabine) OR methotrexate) OR vinblastine) OR doxorubicin) OR platin) OR carboplatin | 203120 |
| #4 | Search ((random*) OR randomized) OR randomised | 1272824 |
| #3 | Search (((advanced) OR metastases) OR metastatic) OR late-stage | 1652303 |
| #2 | Search ((((neoplasms) OR cancer) OR malignant) OR carcinoma) OR tumor | 4331694 |
| #1 | Search (((((bladder) OR intravesical) OR urothelial) OR urethral) OR urothelium) OR urinary | 791737 |
