# Supplementary material for: Efficacy and Safety of Chemotherapy Regimens in Advanced or Metastatic Bladder and Urothelial Carcinomas: An Updated Network Meta-Analysis
Source: Front Pharmacol. 2020 Jan 15;10:1507. doi: 10.3389/fphar.2019.01507 (PMC6974923; doi:10.3389/fphar.2019.01507)
Supplement: Supplementary Table 2 — The PFS results of the chemotherapy strategies according to their relative effect and reliable quality. [file Table_2.docx]

Supplementary table 2. The PFS results of chemotherapy strategies according to their relative effect and reliable quality.

| Interventions | | Direct comparisons | | Indirect comparisons | | Network comparisons | |
| --- | --- | --- | --- | --- | --- | --- | --- |
|  |  | LogHR(95%CIs) | Quality | LogHR(95%CIs) | Quality | LogHR(95%CIs) | Quality |
| GCA vs. |  |  |  |  |  |  |  |
|  | PGC |  |  | 0.03 (-0.31,0.37) | Moderate* | 0.03 (-0.31,0.37) | Moderate* |
|  | PC |  |  | **-0.92 (-1.58,-0.26)** | Low*‡ | **-0.92 (-1.58,-0.26)** | Low*‡ |
|  | MVAC |  |  | -0.00 (-0.36,0.35) | Moderate* | -0.00 (-0.36,0.35) | Moderate* |
|  | MCAVI |  |  | -0.44 (-0.92,0.05) | Moderate* | -0.44 (-0.92,0.05) | Moderate* |
|  | LC |  |  | **-0.64 (-1.07,-0.22)** | Low*‡ | **-0.64 (-1.07,-0.22)** | Low*‡ |
|  | GCS |  |  | -0.11 (-0.64,0.42) | Moderate‡ | -0.11 (-0.64,0.42) | Moderate‡ |
|  | GP |  |  | -0.36 (-0.81,0.09) | Low*‡ | -0.36 (-0.81,0.09) | Low*‡ |
|  | GCG |  |  | -0.20 (-0.57,0.18) | Moderate* | -0.20 (-0.57,0.18) | Moderate* |
|  | GC | -0.13 (-0.44,0.18) | High | NA | NA | -0.13 (-0.44,0.18) | **High** |
|  | FAP |  |  | -0.24 (-0.72,0.24) | Low*‡ | -0.24 (-0.72,0.24) | Low*‡ |
|  | DC |  |  | **-0.55 (-1.04,-0.07)** | **Low*‡** | **-0.55 (-1.04,-0.07)** | **Low*‡** |
|  | CP |  |  | -0.26 (-0.85,0.33) | Low*‡ | -0.26 (-0.85,0.33) | Low*‡ |
|  | CIS |  |  | **-0.79 (-1.23,-0.35)** | **Low*‡** | **-0.79 (-1.23,-0.35)** | **Low*‡** |
|  | GCCET |  |  | -0.20 (-0.75,0.36) | Low*‡ | -0.20 (-0.75,0.36) | Low*‡ |
| PGC vs. |  |  |  |  |  |  |  |
|  | PC |  |  | **-0.95 (-1.56,-0.35)** | **Low*‡** | **-0.95 (-1.56,-0.35)** | **Low*‡** |
|  | MVAC |  |  | -0.03 (-0.27,0.20) | Moderate* | -0.03 (-0.27,0.20) | Moderate* |
|  | MCAVI |  |  | **-0.47 (-0.87,-0.06)** | **Moderate*** | **-0.47 (-0.87,-0.06)** | **Moderate*** |
|  | LC |  |  | **-0.67 (-1.01,-0.34)** | **Moderate*** | **-0.67 (-1.01,-0.34)** | **Moderate*** |
|  | GCS |  |  | -0.14 (-0.60,0.32) | Low*‡ | -0.14 (-0.60,0.32) | Low*‡ |
|  | GP |  |  | **-0.39 (-0.76,-0.02)** | **Moderate*** | **-0.39 (-0.76,-0.02)** | **Moderate*** |
|  | GCG |  |  | -0.23 (-0.50,0.05) | Moderate* | -0.23 (-0.50,0.05) | Moderate* |
|  | GC | **-0.16 (-0.32,-0.00)** | **Moderate*** |  |  | **-0.16 (-0.32,-0.00)** | **Moderate*** |
|  | FAP |  |  | -0.27 (-0.67,0.13) | Low*‡ | -0.27 (-0.67,0.13) | Low*‡ |
|  | DC |  |  | **-0.58 (-0.99,-0.17)** | **Low*‡** | **-0.58 (-0.99,-0.17)** | **Low*‡** |
|  | CP |  |  | -0.29 (-0.82,0.24) | Low*‡ | -0.29 (-0.82,0.24) | Low*‡ |
|  | CIS |  |  | **-0.82 (-1.17,-0.46)** | **High*†** | **-0.82 (-1.17,-0.46)** | **High*†** |
|  | GCCET |  |  | -0.23 (-0.71,0.26) | Low*‡ | -0.23 (-0.71,0.26) | Low*‡ |
| PC vs. |  |  |  |  |  |  |  |
|  | MVAC |  |  | **0.92 (0.31,1.53)** | **Low*‡** | **0.92 (0.31,1.53)** | **Low*‡** |
|  | MCAVI |  |  | 0.49 (-0.21,1.18) | Low*‡ | 0.49 (-0.21,1.18) | Low*‡ |
|  | LC |  |  | 0.28 (-0.38,0.94) | Low*‡ | 0.28 (-0.38,0.94) | Low*‡ |
|  | GCS |  |  | **0.82 (0.09,1.55)** | **Low*‡** | **0.82 (0.09,1.55)** | **Low*‡** |
|  | GP |  |  | 0.56 (-0.11,1.23) | Low*‡ | 0.56 (-0.11,1.23) | Low*‡ |
|  | GCG |  |  | **0.73 (0.10,1.35)** | **Low*‡** | **0.73 (0.10,1.35)** | **Low*‡** |
|  | GC | **0.79 (0.21,1.38)** | **Low*‡** |  |  | **0.79 (0.21,1.38)** | **Low*‡** |
|  | FAP |  |  | 0.68 (-0.01,1.37) | Low*‡ | 0.68 (-0.01,1.37) | Low*‡ |
|  | DC |  |  | 0.37 (-0.33,1.07) | Low*‡ | 0.37 (-0.33,1.07) | Low*‡ |
|  | CP |  |  | 0.66 (-0.11,1.44) | Low*‡ | 0.66 (-0.11,1.44) | Low*‡ |
|  | CIS |  |  | 0.13 (-0.53,0.80) | Low*‡ | 0.13 (-0.53,0.80) | Low*‡ |
|  | GCCET |  |  | 0.72 (-0.02,1.47) | Low*‡ | 0.72 (-0.02,1.47) | Low*‡ |
| MVAC vs. |  |  |  |  |  |  |  |
|  | MCAVI | **-0.63(-1.26,-0.01)** | Low*‡ | -0.30(-0.80,0.19) | Low*‡ | **-0.43 (-0.82,-0.05)** | **Low*#** |
|  | LC |  |  | **-0.64 (-0.98,-0.29)** | Moderate* | **-0.64 (-0.98,-0.29)** | Moderate* |
|  | GCS |  |  | -0.10 (-0.57,0.37) | Low*‡ | -0.10 (-0.57,0.37) | Low*‡ |
|  | GP |  |  | **-0.36 (-0.71,-0.00)** | Moderate* | **-0.36 (-0.71,-0.00)** | Moderate* |
|  | GCG |  |  | -0.19 (-0.47,0.09) | Moderate* | -0.19 (-0.47,0.09) | Moderate* |
|  | GC | -0.11(-0.29,0.08) | Moderate* | -0.44(-1.21,0.33) | Low*‡ | -0.12 (-0.30,0.05) | Moderate* |
|  | FAP | -0.24 (-0.56,0.09) | Moderate* | NA | NA | -0.24 (-0.56,0.09) | Moderate* |
|  | DC | **-0.55 (-0.88,-0.21)** | **Moderate*** | NA | NA | **-0.55 (-0.88,-0.21)** | **Moderate*** |
|  | CP | -0.25 (-0.73,0.22) | Low*‡ | NA | NA | -0.25 (-0.73,0.22) | Low*‡ |
|  | CIS | **-0.78 (-1.05,-0.52)** | Moderate* | NA | NA | **-0.78 (-1.05,-0.52)** | Moderate* |
|  | GCCET |  |  | -0.19 (-0.69,0.30) | Low*‡ | -0.19 (-0.69,0.30) | Low*‡ |
| MCAVI vs. | |  |  |  |  |  |  |
|  | LC |  |  | -0.21 (-0.68,0.27) | Low*‡ | -0.21 (-0.68,0.27) | Low*‡ |
|  | GCS |  |  | 0.33 (-0.24,0.90) | Low*‡ | 0.33 (-0.24,0.90) | Low*‡ |
|  | GP | 0.04(-0.22,0.30) | Moderate* | 0.37(-0.38,1.12) | Low*‡ | 0.08 (-0.17,0.32) | Moderate* |
|  | GCG |  |  | 0.24 (-0.19,0.68) | Low*‡ | 0.24 (-0.19,0.68) | Low*‡ |
|  | GC |  |  | 0.31 (-0.07,0.68) | Moderate* | 0.31 (-0.07,0.68) | Moderate* |
|  | FAP |  |  | 0.20 (-0.31,0.70) | Low*‡ | 0.20 (-0.31,0.70) | Low*‡ |
|  | DC |  |  | -0.12 (-0.63,0.39) | Low*‡ | -0.12 (-0.63,0.39) | Low*‡ |
|  | CP |  |  | 0.18 (-0.44,0.79) | Low*‡ | 0.18 (-0.44,0.79) | Low*‡ |
|  | CIS |  |  | -0.35 (-0.82,0.12) | Low*‡ | -0.35 (-0.82,0.12) | Low*‡ |
|  | GCCET |  |  | 0.24 (-0.35,0.83) | Low*‡ | 0.24 (-0.35,0.83) | Low*‡ |
| LC vs. |  |  |  |  |  |  |  |
|  | GCS |  |  | **0.54 (0.01,1.06)** | **Low*‡** | **0.54 (0.01,1.06)** | **Low*‡** |
|  | GP |  |  | 0.28 (-0.17,0.73) | Low*‡ | 0.28 (-0.17,0.73) | Low*‡ |
|  | GCG |  |  | **0.45 (0.08,0.82)** | **Moderate*** | **0.45 (0.08,0.82)** | **Moderate*** |
|  | GC | **0.51 (0.21,0.81)** | **Moderate*** | NA | NA | **0.51 (0.21,0.81)** | **Moderate*** |
|  | FAP |  |  | 0.40 (-0.07,0.87) | Low*‡ | 0.40 (-0.07,0.87) | Low*‡ |
|  | DC |  |  | 0.09 (-0.39,0.57) | Low*‡ | 0.09 (-0.39,0.57) | Low*‡ |
|  | CP |  |  | 0.38 (-0.21,0.97) | Low*‡ | 0.38 (-0.21,0.97) | Low*‡ |
|  | CIS |  |  | -0.15 (-0.58,0.29) | Low*‡ | -0.15 (-0.58,0.29) | Low*‡ |
|  | GCCET |  |  | 0.45 (-0.10,0.99) | Low*‡ | 0.45 (-0.10,0.99) | Low*‡ |
| GCS vs. |  |  |  |  |  |  |  |
|  | GP |  |  | -0.26 (-0.80,0.29) | Low*‡ | -0.26 (-0.80,0.29) | Low*‡ |
|  | GCG |  |  | -0.09 (-0.58,0.40) | Low*‡ | -0.09 (-0.58,0.40) | Low*‡ |
|  | GC | -0.02 (-0.46,0.41) | Moderate‡ | NA | NA | -0.02 (-0.46,0.41) | Moderate‡ |
|  | FAP |  |  | -0.13 (-0.70,0.43) | Low*‡ | -0.13 (-0.70,0.43) | Low*‡ |
|  | DC |  |  | -0.45 (-1.02,0.13) | Low*‡ | -0.45 (-1.02,0.13) | Low*‡ |
|  | CP |  |  | -0.15 (-0.82,0.52) | Low*‡ | -0.15 (-0.82,0.52) | Low*‡ |
|  | CIS |  |  | **-0.68 (-1.22,-0.14)** | Low*‡ | **-0.68 (-1.22,-0.14)** | Low*‡ |
|  | GCCET |  |  | -0.09 (-0.72,0.54) | Low*‡ | -0.09 (-0.72,0.54) | Low*‡ |
| GP vs. |  |  |  |  |  |  |  |
|  | GCG |  |  | 0.17 (-0.23,0.56) | Low*‡ | 0.17 (-0.23,0.56) | Low*‡ |
|  | GC | 0.16(-0.22,0.54) | Moderate* | 0.49(-0.21,1.19) | Low*‡ | 0.23 (-0.10,0.56) | Moderate* |
|  | FAP |  |  | 0.12 (-0.36,0.60) | Low*‡ | 0.12 (-0.36,0.60) | Low*‡ |
|  | DC |  |  | -0.19 (-0.68,0.30) | Low*‡ | -0.19 (-0.68,0.30) | Low*‡ |
|  | CP |  |  | 0.10 (-0.49,0.70) | Low*‡ | 0.10 (-0.49,0.70) | Low*‡ |
|  | CIS |  |  | -0.43 (-0.87,0.02) | Low*‡ | -0.43 (-0.87,0.02) | Low*‡ |
|  | GCCET |  |  | 0.16 (-0.40,0.73) | Low*‡ | 0.16 (-0.40,0.73) | Low*‡ |
| GCG vs. |  |  |  |  |  |  |  |
|  | GC | 0.07 (-0.16,0.29) | Moderate* | NA | NA | 0.07 (-0.16,0.29) | Moderate* |
|  | FAP |  |  | -0.04 (-0.47,0.38) | Low*‡ | -0.04 (-0.47,0.38) | Low*‡ |
|  | DC |  |  | -0.36 (-0.80,0.08) | Low*‡ | -0.36 (-0.80,0.08) | Low*‡ |
|  | CP |  |  | -0.06 (-0.62,0.49) | Low*‡ | -0.06 (-0.62,0.49) | Low*‡ |
|  | CIS |  |  | **-0.59 (-0.98,-0.20)** | **Moderate*** | **-0.59 (-0.98,-0.20)** | **Moderate*** |
|  | GCCET |  |  | -0.00 (-0.51,0.51) | Low*‡ | -0.00 (-0.51,0.51) | Low*‡ |
| GC vs. |  |  |  |  |  |  |  |
|  | FAP |  |  | -0.11 (-0.48,0.26) | Moderate* | -0.11 (-0.48,0.26) | Moderate* |
|  | DC |  |  | **-0.42 (-0.80,-0.05)** | **Moderate*** | **-0.42 (-0.80,-0.05)** | **Moderate*** |
|  | CP |  |  | -0.13 (-0.64,0.38) | Low*‡ | -0.13 (-0.64,0.38) | Low*‡ |
|  | CIS |  |  | **-0.66 (-0.98,-0.34)** | **Moderate*** | **-0.66 (-0.98,-0.34)** | **Moderate*** |
|  | GCCET | -0.07 (-0.53,0.39) | Low*‡ | NA | NA | -0.07 (-0.53,0.39) | Low*‡ |
| FAP vs. |  |  |  |  |  |  |  |
|  | DC |  |  | -0.31 (-0.78,0.15) | Low*‡ | -0.31 (-0.78,0.15) | Low*‡ |
|  | CP |  |  | -0.02 (-0.59,0.56) | Low*‡ | -0.02 (-0.59,0.56) | Low*‡ |
|  | CIS |  |  | **-0.55 (-0.96,-0.13)** | **Low*‡** | **-0.55 (-0.96,-0.13)** | **Low*‡** |
|  | GCCET |  |  | 0.04 (-0.55,0.63) | Low*‡ | 0.04 (-0.55,0.63) | Low*‡ |
| DC vs. |  |  |  |  |  |  |  |
|  | CP |  |  | 0.29 (-0.29,0.88) | Low*‡ | 0.29 (-0.29,0.88) | Low*‡ |
|  | CIS |  |  | -0.24 (-0.66,0.19) | Low*‡ | -0.24 (-0.66,0.19) | Low*‡ |
|  | GCCET |  |  | 0.36 (-0.24,0.95) | Low*‡ | 0.36 (-0.24,0.95) | Low*‡ |
| CP vs. |  |  |  |  |  |  |  |
|  | CIS |  |  | -0.53 (-1.08,0.02) | Low*‡ | -0.53 (-1.08,0.02) | Low*‡ |
|  | GCCET |  |  | 0.06 (-0.62,0.75) | Low*‡ | 0.06 (-0.62,0.75) | Low*‡ |
| CIS vs. |  |  |  |  |  |  |  |
|  | GCCET |  |  | **0.59 (0.03,1.15)** | **Low*‡** | **0.59 (0.03,1.15)** | **Low*‡** |

Abbreviations: CIs: confidence intervals; LogOR: logarithm hazard ratio; NA: not available.

Abbreviations of intervention are showed in Table 1.

Bold means statistic difference (p<0.05).

*: Study limitation; †: Large-scale effect; ‡: Imprecision; #: Incoherence.
