# Supplementary material for: Efficacy and Safety of Chemotherapy Regimens in Advanced or Metastatic Bladder and Urothelial Carcinomas: An Updated Network Meta-Analysis
Source: Front Pharmacol. 2020 Jan 15;10:1507. doi: 10.3389/fphar.2019.01507 (PMC6974923; doi:10.3389/fphar.2019.01507)
Supplement: Supplementary Table 3 — The OS results of the chemotherapy strategies according to their relative effect and reliable quality. [file Table_3.docx]

Supplementary table 3. The OS results of chemotherapy strategies according to their relative effect and reliable quality.

| Interventions | | Direct comparisons | | Indirect comparisons | | Network comparisons | |
| --- | --- | --- | --- | --- | --- | --- | --- |
|  |  | LogHR(95%CIs) | Quality | LogHR(95%CIs) | Quality | LogHR(95%CIs) | Quality |
| CCA vs. |  |  |  |  |  |  |  |
|  | PGC |  |  | **0.90 (0.39,1.42)** | **Low*‡** | **0.90 (0.39,1.42)** | **Low*‡** |
|  | PC |  |  | 0.20 (-0.59,0.99) | Low*‡ | 0.20 (-0.59,0.99) | Low*‡ |
|  | MVAC | **0.80 (0.35,1.25)** | **Low*‡** | NA | NA | **0.80 (0.35,1.25)** | **Low*‡** |
|  | MCAVI |  |  | -0.60 (-1.36,0.16) | Low*‡ | -0.60 (-1.36,0.16) | Low*‡ |
|  | LC |  |  | 0.54 (-0.07,1.16) | Low*‡ | 0.54 (-0.07,1.16) | Low*‡ |
|  | GCS |  |  | **1.04 (0.37,1.70)** | **Low*‡** | **1.04 (0.37,1.70)** | **Low*‡** |
|  | GP |  |  | -0.66 (-1.38,0.05) | Low*‡ | -0.66 (-1.38,0.05) | Low*‡ |
|  | GCG |  |  | **0.59 (0.05,1.13)** | **Low*‡** | **0.59 (0.05,1.13)** | **Low*‡** |
|  | GCCET |  |  | **0.71 (0.03,1.39)** | **Low*‡** | **0.71 (0.03,1.39)** | **Low*‡** |
|  | GC |  |  | **0.73 (0.25,1.22)** | **Low*‡** | **0.73 (0.25,1.22)** | **Low*‡** |
|  | GCA |  |  | **0.86 (0.28,1.45)** | **Low*‡** | **0.86 (0.28,1.45)** | **Low*‡** |
|  | GF |  |  | 0.88 (-0.03,1.79) | Low*‡ | 0.88 (-0.03,1.79) | Low*‡ |
|  | DC |  |  | 0.39 (-0.16,0.93) | Low*‡ | 0.39 (-0.16,0.93) | Low*‡ |
|  | CP |  |  | 0.64 (-0.01,1.29) | Low*‡ | 0.64 (-0.01,1.29) | Low*‡ |
|  | CIS |  |  | 0.29 (-0.23,0.81) | Low*‡ | 0.29 (-0.23,0.81) | Low*‡ |
| PGC vs. |  |  |  |  |  |  |  |
|  | PC |  |  | **-0.70 (-1.34,-0.06)** | **Low*‡** | **-0.70 (-1.34,-0.06)** | **Low*‡** |
|  | MVAC |  |  | -0.10 (-0.35,0.15) | Moderate* | -0.10 (-0.35,0.15) | Moderate* |
|  | MCAVI |  |  | **-1.50 (-2.11,-0.90)** | **Low*‡** | **-1.50 (-2.11,-0.90)** | **Low*‡** |
|  | LC |  |  | -0.36 (-0.77,0.05) | Low*‡ | -0.36 (-0.77,0.05) | Low*‡ |
|  | GCS |  |  | 0.13 (-0.34,0.61) | Low*‡ | 0.13 (-0.34,0.61) | Low*‡ |
|  | GP |  |  | **-1.57 (-2.11,-1.02)** | **Low*‡** | **-1.57 (-2.11,-1.02)** | **Low*‡** |
|  | GCG |  |  | **-0.31 (-0.59,-0.03)** | **Moderate*** | **-0.31 (-0.59,-0.03)** | **Moderate*** |
|  | GCCET |  |  | -0.19 (-0.69,0.31) | Low*‡ | -0.19 (-0.69,0.31) | Low*‡ |
|  | GC | **-0.17 (-0.33,-0.00)** | **Moderate*** | NA | NA | **-0.17 (-0.33,-0.00)** | **Moderate*** |
|  | GCA |  |  | -0.04 (-0.40,0.32) | Moderate* | -0.04 (-0.40,0.32) | Moderate* |
|  | GF |  |  | -0.02 (-0.85,0.80) | Low*‡ | -0.02 (-0.85,0.80) | Low*‡ |
|  | DC |  |  | **-0.52 (-0.92,-0.12)** | **Low*‡** | **-0.52 (-0.92,-0.12)** | **Low*‡** |
|  | CP |  |  | -0.26 (-0.80,0.27) | Low*‡ | -0.26 (-0.80,0.27) | Low*‡ |
|  | CIS |  |  | **-0.61 (-0.97,-0.25)** | **Moderate*** | **-0.61 (-0.97,-0.25)** | **Moderate*** |
| PC vs. |  |  |  |  |  |  |  |
|  | MVAC |  |  | 0.60 (-0.05,1.25) | Low*‡ | 0.60 (-0.05,1.25) | Low*‡ |
|  | MCAVI |  |  | -0.81 (-1.66,0.05) | Low*‡ | -0.81 (-1.66,0.05) | Low*‡ |
|  | LC |  |  | 0.34 (-0.38,1.06) | Low*‡ | 0.34 (-0.38,1.06) | Low*‡ |
|  | GCS |  |  | **0.83 (0.07,1.60)** | **Low*‡** | **0.83 (0.07,1.60)** | **Low*‡** |
|  | GP |  |  | **-0.87 (-1.68,-0.06)** | **Low*‡** | **-0.87 (-1.68,-0.06)** | **Low*‡** |
|  | GCG |  |  | 0.39 (-0.27,1.05) | Low*‡ | 0.39 (-0.27,1.05) | Low*‡ |
|  | GCCET |  |  | 0.51 (-0.27,1.29) | Low*‡ | 0.51 (-0.27,1.29) | Low*‡ |
|  | GC | 0.53 (-0.09,1.15) | Low*‡ | NA | NA | 0.53 (-0.09,1.15) | Low*‡ |
|  | GCA |  |  | 0.66 (-0.04,1.36) | Low*‡ | 0.66 (-0.04,1.36) | Low*‡ |
|  | GF |  |  | 0.68 (-0.34,1.70) | Low*‡ | 0.68 (-0.34,1.70) | Low*‡ |
|  | DC |  |  | 0.18 (-0.54,0.90) | Low*‡ | 0.18 (-0.54,0.90) | Low*‡ |
|  | CP |  |  | 0.44 (-0.36,1.24) | Low*‡ | 0.44 (-0.36,1.24) | Low*‡ |
|  | CIS |  |  | 0.09 (-0.61,0.78) | Low*‡ | 0.09 (-0.61,0.78) | Low*‡ |
| MVAC vs. |  |  |  |  |  |  |  |
|  | MCAVI |  |  | **-1.41 (-2.02,-0.79)** | **Low*‡** | **-1.41 (-2.02,-0.79)** | **Low*‡** |
|  | LC |  |  | -0.26 (-0.68,0.16) | Low*‡ | -0.26 (-0.68,0.16) | Low*‡ |
|  | GCS |  |  | 0.23 (-0.25,0.72) | Low*‡ | 0.23 (-0.25,0.72) | Low*‡ |
|  | GP |  |  | **-1.47 (-2.02,-0.91)** | **Low*‡** | **-1.47 (-2.02,-0.91)** | **Low*‡** |
|  | GCG |  |  | -0.21 (-0.50,0.08) | Moderate* | -0.21 (-0.50,0.08) | Moderate* |
|  | GCCET |  |  | -0.09 (-0.60,0.42) | Low*‡ | -0.09 (-0.60,0.42) | Low*‡ |
|  | GC | -0.07 (-0.26,0.12) | Moderate* | NA | NA | -0.07 (-0.26,0.12) | Moderate* |
|  | GCA |  |  | 0.06 (-0.32,0.43) | Moderate* | 0.06 (-0.32,0.43) | Moderate* |
|  | GF | 0.08 (-0.71,0.86) | Low*‡ | NA | NA | 0.08 (-0.71,0.86) | Low*‡ |
|  | DC | **-0.42 (-0.73,-0.10)** | **Moderate*** | NA | NA | **-0.42 (-0.73,-0.10)** | **Moderate*** |
|  | CP | -0.16 (-0.64,0.31) | Low*‡ | NA | NA | -0.16 (-0.64,0.31) | Low*‡ |
|  | CIS | **-0.51 (-0.77,-0.26)** | **Moderate*** | NA | NA | **-0.51 (-0.77,-0.26)** | **Moderate*** |
| MCAVI vs. |  |  |  |  |  |  |  |
|  | LC |  |  | **1.15 (0.45,1.84)** | **Low*‡** | **1.15 (0.45,1.84)** | **Low*‡** |
|  | GCS |  |  | **1.64 (0.90,2.38)** | **Low*‡** | **1.64 (0.90,2.38)** | **Low*‡** |
|  | GP | -0.06 (-0.33,0.20) | Moderate* | NA | NA | -0.06 (-0.33,0.20) | Moderate* |
|  | GCG |  |  | **1.19 (0.57,1.82)** | **Low*‡** | **1.19 (0.57,1.82)** | **Low*‡** |
|  | GCCET |  |  | **1.32 (0.57,2.07)** | **Low*‡** | **1.32 (0.57,2.07)** | **Low*‡** |
|  | GC |  |  | **1.34 (0.75,1.92)** | **Low*‡** | **1.34 (0.75,1.92)** | **Low*‡** |
|  | GCA |  |  | **1.46 (0.80,2.13)** | **Low*‡** | **1.46 (0.80,2.13)** | **Low*‡** |
|  | GF |  |  | **1.48 (0.48,2.48)** | **Low*‡** | **1.48 (0.48,2.48)** | **Low*‡** |
|  | DC |  |  | **0.99 (0.30,1.68)** | **Low*‡** | **0.99 (0.30,1.68)** | **Low*‡** |
|  | CP |  |  | **1.24 (0.47,2.02)** | **Low*‡** | **1.24 (0.47,2.02)** | **Low*‡** |
|  | CIS |  |  | **0.90 (0.23,1.56)** | **Low*‡** | **0.90 (0.23,1.56)** | **Low*‡** |
| LC vs. |  |  |  |  |  |  |  |
|  | GCS |  |  | 0.49 (-0.09,1.08) | Low*‡ | 0.49 (-0.09,1.08) | Low*‡ |
|  | GP |  |  | **-1.21 (-1.85,-0.57)** | **Low*‡** | **-1.21 (-1.85,-0.57)** | **Low*‡** |
|  | GCG |  |  | 0.05 (-0.39,0.49) | Low*‡ | 0.05 (-0.39,0.49) | Low*‡ |
|  | GCCET |  |  | 0.17 (-0.43,0.77) | Low*‡ | 0.17 (-0.43,0.77) | Low*‡ |
|  | GC | 0.19 (-0.19,0.57) | Moderate* | NA | NA | 0.19 (-0.19,0.57) | Moderate* |
|  | GCA |  |  | 0.32 (-0.18,0.81) | Low*‡ | 0.32 (-0.18,0.81) | Low*‡ |
|  | GF |  |  | 0.34 (-0.56,1.23) | Moderate* | 0.34 (-0.56,1.23) | Moderate* |
|  | DC |  |  | -0.16 (-0.68,0.37) | Low*‡ | -0.16 (-0.68,0.37) | Low*‡ |
|  | CP |  |  | 0.10 (-0.54,0.73) | Low*‡ | 0.10 (-0.54,0.73) | Low*‡ |
|  | CIS |  |  | -0.25 (-0.74,0.24) | Low*‡ | -0.25 (-0.74,0.24) | Low*‡ |
| GCS vs. |  |  |  |  |  |  |  |
|  | GP |  |  | **-1.70 (-2.39,-1.01)** | **Low*‡** | **-1.70 (-2.39,-1.01)** | **Low*‡** |
|  | GCG |  |  | -0.45 (-0.95,0.06) | Low*‡ | -0.45 (-0.95,0.06) | Low*‡ |
|  | GCCET |  |  | -0.32 (-0.97,0.33) | Low*‡ | -0.32 (-0.97,0.33) | Low*‡ |
|  | GC | -0.30 (-0.75,0.14) | Moderate‡ | NA | NA | -0.30 (-0.75,0.14) | Moderate‡ |
|  | GCA |  |  | -0.18 (-0.73,0.38) | Moderate‡ | -0.18 (-0.73,0.38) | Moderate‡ |
|  | GF |  |  | -0.16 (-1.08,0.77) | Low*‡ | -0.16 (-1.08,0.77) | Low*‡ |
|  | DC |  |  | **-0.65 (-1.23,-0.07)** | **Low*‡** | **-0.65 (-1.23,-0.07)** | **Low*‡** |
|  | CP |  |  | -0.40 (-1.07,0.28) | Low*‡ | -0.40 (-1.07,0.28) | Low*‡ |
|  | CIS |  |  | **-0.74 (-1.29,-0.20)** | **Low*‡** | **-0.74 (-1.29,-0.20)** | **Low*‡** |
| GP vs. |  |  |  |  |  |  |  |
|  | GCG |  |  | **1.26 (0.69,1.82)** | **Low*‡** | **1.26 (0.69,1.82)** | **Low*‡** |
|  | GCCET |  |  | **1.38 (0.68,2.08)** | **Low*‡** | **1.38 (0.68,2.08)** | **Low*‡** |
|  | GC | **1.40 (0.88,1.92)** | **Low*‡** | NA | NA | **1.40 (0.88,1.92)** | **Low*‡** |
|  | GCA |  |  | **1.53 (0.91,2.14)** | **Low*‡** | **1.53 (0.91,2.14)** | **Low*‡** |
|  | GF |  |  | **1.54 (0.58,2.51)** | **Low*‡** | **1.54 (0.58,2.51)** | **Low*‡** |
|  | DC |  |  | **1.05 (0.41,1.69)** | **Low*‡** | **1.05 (0.41,1.69)** | **Low*‡** |
|  | CP |  |  | **1.31 (0.58,2.03)** | **Low*‡** | **1.31 (0.58,2.03)** | **Low*‡** |
|  | CIS |  |  | **0.96 (0.35,1.57)** | **Low*‡** | **0.96 (0.35,1.57)** | **Low*‡** |
| GCG vs. |  |  |  |  |  |  |  |
|  | GCCET |  |  | 0.12 (-0.40,0.64) | Low*‡ | 0.12 (-0.40,0.64) | Low*‡ |
|  | GC | 0.14 (-0.08,0.37) | Moderate* | NA | NA | 0.14 (-0.08,0.37) | Moderate* |
|  | GCA |  |  | 0.27 (-0.12,0.66) | Moderate* | 0.27 (-0.12,0.66) | Moderate* |
|  | GF |  |  | 0.29 (-0.55,1.13) | Low*‡ | 0.29 (-0.55,1.13) | Low*‡ |
|  | DC |  |  | -0.21 (-0.64,0.22) | Low*‡ | -0.21 (-0.64,0.22) | Low*‡ |
|  | CP |  |  | 0.05 (-0.51,0.60) | Low*‡ | 0.05 (-0.51,0.60) | Low*‡ |
|  | CIS |  |  | -0.30 (-0.69,0.09) | Moderate* | -0.30 (-0.69,0.09) | Moderate* |
| GCCET vs. |  |  |  |  |  |  |  |
|  | GC | 0.02 (-0.45,0.49) | Low*‡ | NA | NA | 0.02 (-0.45,0.49) | Low*‡ |
|  | GCA |  |  | 0.15 (-0.42,0.72) | Low*‡ | 0.15 (-0.42,0.72) | Low*‡ |
|  | GF |  |  | 0.17 (-0.77,1.10) | Low*‡ | 0.17 (-0.77,1.10) | Low*‡ |
|  | DC |  |  | -0.33 (-0.92,0.27) | Low*‡ | -0.33 (-0.92,0.27) | Low*‡ |
|  | CP |  |  | -0.07 (-0.77,0.62) | Low*‡ | -0.07 (-0.77,0.62) | Low*‡ |
|  | CIS |  |  | -0.42 (-0.99,0.15) | Low*‡ | -0.42 (-0.99,0.15) | Low*‡ |
| GC vs. |  |  |  |  |  |  |  |
|  | GCA | 0.13 (-0.20,0.45) | High | NA | NA | 0.13 (-0.20,0.45) | high |
|  | GF |  |  | 0.15 (-0.66,0.96) | Low*‡ | 0.15 (-0.66,0.96) | Low*‡ |
|  | DC |  |  | -0.35 (-0.71,0.02) | Moderate* | -0.35 (-0.71,0.02) | Moderate* |
|  | CP |  |  | -0.09 (-0.60,0.42) | Low*‡ | -0.09 (-0.60,0.42) | Low*‡ |
|  | CIS |  |  | **-0.44 (-0.76,-0.12)** | **Moderate*** | **-0.44 (-0.76,-0.12)** | **Moderate*** |
| GCA vs. |  |  |  |  |  |  |  |
|  | GF |  |  | 0.02 (-0.85,0.89) | Low*‡ | 0.02 (-0.85,0.89) | Low*‡ |
|  | DC |  |  | -0.48 (-0.96,0.01) | Low*‡ | -0.48 (-0.96,0.01) | Low*‡ |
|  | CP |  |  | -0.22 (-0.82,0.38) | Low*‡ | -0.22 (-0.82,0.38) | Low*‡ |
|  | CIS |  |  | **-0.57 (-1.02,-0.12)** | **Low*‡** | **-0.57 (-1.02,-0.12)** | **Low*‡** |
| GF vs. |  |  |  |  |  |  |  |
|  | DC |  |  | -0.49 (-1.34,0.35) | Low*‡ | -0.49 (-1.34,0.35) | Low*‡ |
|  | CP |  |  | -0.24 (-1.16,0.68) | Low*‡ | -0.24 (-1.16,0.68) | Low*‡ |
|  | CIS |  |  | -0.59 (-1.42,0.24) | Low*‡ | -0.59 (-1.42,0.24) | Low*‡ |
| DC vs. |  |  |  |  |  |  |  |
|  | CP |  |  | 0.26 (-0.31,0.82) | Low*‡ | 0.26 (-0.31,0.82) | Low*‡ |
|  | CIS |  |  | -0.09 (-0.50,0.31) | Low*‡ | -0.09 (-0.50,0.31) | Low*‡ |
| CP vs. |  |  |  |  |  |  |  |
|  | CIS |  |  | -0.35 (-0.89,0.19) | Low*‡ | -0.35 (-0.89,0.19) | Low*‡ |

Abbreviations: CIs: confidence intervals; LogOR: logarithm hazard ratio; NA: not available.

Abbreviations of intervention are showed in Table 1.

Bold means statistic difference (p<0.05).

*: Study limitation; †: Large-scale effect; ‡: Imprecision; #: Incoherence.
