# Supplementary material for: Efficacy and Safety of Chemotherapy Regimens in Advanced or Metastatic Bladder and Urothelial Carcinomas: An Updated Network Meta-Analysis
Source: Front Pharmacol. 2020 Jan 15;10:1507. doi: 10.3389/fphar.2019.01507 (PMC6974923; doi:10.3389/fphar.2019.01507)
Supplement: Supplementary Table 4 — The ORR results of the chemotherapy strategies according to their relative effect and reliable quality. [file Table_4.docx]

Supplementary table 4. The ORR results of chemotherapy strategies according to their relative effect and reliable quality.

| Interventions | | Direct comparisons | | Indirect comparisons | | Network comparisons | |
| --- | --- | --- | --- | --- | --- | --- | --- |
|  |  | logOR(95%CIs) | Quality | logOR(95%CIs) | Quality | logOR(95%CIs) | Quality |
| CCA vs. |  |  |  |  |  |  |  |
|  | PGC |  |  | **-1.37 (-2.35,-0.39)** | Low*‡ | **-1.37 (-2.35,-0.39)** | Low*‡ |
|  | PC |  |  | 0.03 (-1.42,1.49) | Low*‡ | 0.03 (-1.42,1.49) | Low*‡ |
|  | MVAC | -0.78 (-1.57,0.02) | Low*‡ | NA | NA | -0.78 (-1.57,0.02) | Low*‡ |
|  | MCAVI |  |  | -0.15 (-1.30,0.99) | Low*‡ | -0.15 (-1.30,0.99) | Low*‡ |
|  | LC |  |  | -0.45 (-1.56,0.65) | Low*‡ | -0.45 (-1.56,0.65) | Low*‡ |
|  | GP |  |  | -0.61 (-1.75,0.52) | Low*‡ | -0.61 (-1.75,0.52) | Low*‡ |
|  | GCS |  |  | -1.18 (-2.43,0.07) | Low*‡ | -1.18 (-2.43,0.07) | Low*‡ |
|  | GCG |  |  | **-1.35 (-2.61,-0.09)** | Low*‡ | **-1.35 (-2.61,-0.09)** | Low*‡ |
|  | GCCET |  |  | -1.13 (-2.44,0.18) | Low*‡ | -1.13 (-2.44,0.18) | Low*‡ |
|  | GCA |  |  | -0.66 (-1.78,0.47) | Low*‡ | -0.66 (-1.78,0.47) | Low*‡ |
|  | GC |  |  | **-0.96 (-1.89,-0.02)** | Low*‡ | **-0.96 (-1.89,-0.02)** | Low*‡ |
|  | GF |  |  | **4.01 (1.38,6.63)** | Low*‡ | **4.01 (1.38,6.63)** | Low*‡ |
|  | FAP |  |  | -0.09 (-1.09,0.92) | Low*‡ | -0.09 (-1.09,0.92) | Low*‡ |
|  | DC |  |  | -0.09 (-1.09,0.91) | Low*‡ | -0.09 (-1.09,0.91) | Low*‡ |
|  | CP |  |  | -0.62 (-1.85,0.61) | Low*‡ | -0.62 (-1.85,0.61) | Low*‡ |
|  | CIS |  |  | 0.73 (-0.31,1.76) | Low*‡ | 0.73 (-0.31,1.76) | Low*‡ |
| PGC vs. |  |  |  |  |  |  |  |
|  | PC |  |  | **1.40 (0.24,2.56)** | Low*‡ | **1.40 (0.24,2.56)** | Low*‡ |
|  | MVAC |  |  | **0.59 (0.02,1.16)** | Low*‡ | **0.59 (0.02,1.16)** | Low*‡ |
|  | MCAVI |  |  | **1.21 (0.35,2.08)** | Low*‡ | **1.21 (0.35,2.08)** | Low*‡ |
|  | LC |  |  | **0.91 (0.25,1.58)** | Low*‡ | **0.91 (0.25,1.58)** | Low*‡ |
|  | GP |  |  | 0.75 (-0.06,1.57) | Low*‡ | 0.75 (-0.06,1.57) | Low*‡ |
|  | GCS |  |  | 0.19 (-0.70,1.08) | Low*‡ | 0.19 (-0.70,1.08) | Low*‡ |
|  | GCG |  |  | 0.02 (-0.88,0.92) | Low*‡ | 0.02 (-0.88,0.92) | Low*‡ |
|  | GCCET |  |  | 0.24 (-0.73,1.20) | Low*‡ | 0.24 (-0.73,1.20) | Low*‡ |
|  | GCA |  |  | **0.71 (0.01,1.40)** | Low*‡ | **0.71 (0.01,1.40)** | Low*‡ |
|  | GC | **0.41 (0.12,0.71)** | **Moderate*** | NA | NA | **0.41 (0.12,0.71)** | **Moderate*** |
|  | GF |  |  | **5.38 (2.81,7.94)** | Low*‡ | **5.38 (2.81,7.94)** | Low*‡ |
|  | FAP |  |  | **1.28 (0.45,2.12)** | Low*‡ | **1.28 (0.45,2.12)** | Low*‡ |
|  | DC |  |  | **1.28 (0.44,2.11)** | Low*‡ | **1.28 (0.44,2.11)** | Low*‡ |
|  | CP |  |  | 0.74 (-0.35,1.84) | Low*‡ | 0.74 (-0.35,1.84) | Low*‡ |
|  | CIS |  |  | **2.09 (1.22,2.96)** | Low*‡ | **2.09 (1.22,2.96)** | Low*‡ |
| PC vs. |  |  |  |  |  |  |  |
|  | MVAC |  |  | -0.81 (-2.03,0.41) | Low*‡ | -0.81 (-2.03,0.41) | Low*‡ |
|  | MCAVI |  |  | -0.19 (-1.57,1.20) | Low*‡ | -0.19 (-1.57,1.20) | Low*‡ |
|  | LC |  |  | -0.49 (-1.75,0.78) | Low*‡ | -0.49 (-1.75,0.78) | Low*‡ |
|  | GP |  |  | -0.65 (-2.00,0.71) | Low*‡ | -0.65 (-2.00,0.71) | Low*‡ |
|  | GCS |  |  | -1.21 (-2.61,0.19) | Low*‡ | -1.21 (-2.61,0.19) | Low*‡ |
|  | GCG |  |  | -1.38 (-2.78,0.02) | Low*‡ | -1.38 (-2.78,0.02) | Low*‡ |
|  | GCCET |  |  | -1.16 (-2.61,0.28) | Low*‡ | -1.16 (-2.61,0.28) | Low*‡ |
|  | GCA |  |  | -0.69 (-1.97,0.59) | Low*‡ | -0.69 (-1.97,0.59) | Low*‡ |
|  | GC | -0.99 (-2.11,0.13) | Low*‡ | NA | NA | -0.99 (-2.11,0.13) | Low*‡ |
|  | GF |  |  | **3.98 (1.19,6.76)** | Low*‡ | **3.98 (1.19,6.76)** | Low*‡ |
|  | FAP |  |  | -0.12 (-1.48,1.25) | Low*‡ | -0.12 (-1.48,1.25) | Low*‡ |
|  | DC |  |  | -0.12 (-1.49,1.24) | Low*‡ | -0.12 (-1.49,1.24) | Low*‡ |
|  | CP |  |  | -0.66 (-2.19,0.88) | Low*‡ | -0.66 (-2.19,0.88) | Low*‡ |
|  | CIS |  |  | 0.69 (-0.69,2.08) | Low*‡ | 0.69 (-0.69,2.08) | Low*‡ |
| MVAC vs. |  |  |  |  |  |  |  |
|  | MCAVI | 0.53(-0.64,1.70) | Low*‡ | 0.72(-0.45,1.88) | Low*‡ | 0.62 (-0.20,1.45) | Low*‡ |
|  | LC |  |  | 0.32 (-0.44,1.09) | Low*‡ | 0.32 (-0.44,1.09) | Low*‡ |
|  | GP |  |  | 0.16 (-0.64,0.97) | Low*‡ | 0.16 (-0.64,0.97) | Low*‡ |
|  | GCS |  |  | -0.40 (-1.37,0.57) | Low*‡ | -0.40 (-1.37,0.57) | Low*‡ |
|  | GCG |  |  | -0.57 (-1.55,0.41) | Low*‡ | -0.57 (-1.55,0.41) | Low*‡ |
|  | GCCET |  |  | -0.35 (-1.39,0.68) | Low*‡ | -0.35 (-1.39,0.68) | Low*‡ |
|  | GCA |  |  | 0.12 (-0.67,0.91) | Low*‡ | 0.12 (-0.67,0.91) | Low*‡ |
|  | GC | -0.16(-0.67,0.35) | Low*‡ | -0.35(-1.92,1.22) | Low*‡ | -0.18 (-0.66,0.31) | Low*‡ |
|  | GF | **4.79 (2.29,7.29)** | Low*‡ | NA | NA | **4.79 (2.29,7.29)** | Low*‡ |
|  | FAP | **0.69 (0.08,1.30)** | Low*‡ | NA | NA | **0.69 (0.08,1.30)** | Low*‡ |
|  | DC | **0.69 (0.08,1.29)** | Low*‡ | NA | NA | **0.69 (0.08,1.29)** | Low*‡ |
|  | CP | 0.15 (-0.78,1.09) | Low*‡ | NA | NA | 0.15 (-0.78,1.09) | Low*‡ |
|  | CIS | **1.50 (0.85,2.16)** | Low*‡ | NA | NA | **1.50 (0.85,2.16)** | Low*‡ |
| MCAVI vs. |  |  |  |  |  |  |  |
|  | LC |  |  | -0.30 (-1.30,0.71) | Low*‡ | -0.30 (-1.30,0.71) | Low*‡ |
|  | GP | -0.48(-1.01,0.06) | Low*‡ | -0.29(-1.85,1.27) | Low*‡ | -0.46 (-0.97,0.05) | Low*‡ |
|  | GCS |  |  | -1.02 (-2.19,0.14) | Low*‡ | -1.02 (-2.19,0.14) | Low*‡ |
|  | GCG |  |  | **-1.19 (-2.37,-0.02)** | Low*‡ | **-1.19 (-2.37,-0.02)** | Low*‡ |
|  | GCCET |  |  | -0.98 (-2.20,0.25) | Low*‡ | -0.98 (-2.20,0.25) | Low*‡ |
|  | GCA |  |  | -0.50 (-1.53,0.52) | Low*‡ | -0.50 (-1.53,0.52) | Low*‡ |
|  | GC |  |  | -0.80 (-1.61,0.01) | Low*‡ | -0.80 (-1.61,0.01) | Low*‡ |
|  | GF |  |  | **4.16 (1.53,6.80)** | Low*‡ | **4.16 (1.53,6.80)** | Low*‡ |
|  | FAP |  |  | 0.07 (-0.96,1.10) | Low*‡ | 0.07 (-0.96,1.10) | Low*‡ |
|  | DC |  |  | 0.06 (-0.96,1.09) | Low*‡ | 0.06 (-0.96,1.09) | Low*‡ |
|  | CP |  |  | -0.47 (-1.72,0.78) | Low*‡ | -0.47 (-1.72,0.78) | Low*‡ |
|  | CIS |  |  | 0.88 (-0.18,1.94) | Low*‡ | 0.88 (-0.18,1.94) | Low*‡ |
| LC vs. |  |  |  |  |  |  |  |
|  | GP |  |  | -0.16 (-1.12,0.80) | Low*‡ | -0.16 (-1.12,0.80) | Low*‡ |
|  | GCS |  |  | -0.72 (-1.75,0.30) | Low*‡ | -0.72 (-1.75,0.30) | Low*‡ |
|  | GCG |  |  | -0.89 (-1.93,0.14) | Low*‡ | -0.89 (-1.93,0.14) | Low*‡ |
|  | GCCET |  |  | -0.68 (-1.77,0.42) | Low*‡ | -0.68 (-1.77,0.42) | Low*‡ |
|  | GCA |  |  | -0.20 (-1.07,0.66) | Low*‡ | -0.20 (-1.07,0.66) | Low*‡ |
|  | GC | -0.50 (-1.09,0.09) | Low*‡ | NA | NA | -0.50 (-1.09,0.09) | Low*‡ |
|  | GF |  |  | **4.46 (1.85,7.08)** | Low*‡ | **4.46 (1.85,7.08)** | Low*‡ |
|  | FAP |  |  | 0.37 (-0.61,1.35) | Low*‡ | 0.37 (-0.61,1.35) | Low*‡ |
|  | DC |  |  | 0.36 (-0.61,1.34) | Low*‡ | 0.36 (-0.61,1.34) | Low*‡ |
|  | CP |  |  | -0.17 (-1.38,1.04) | Low*‡ | -0.17 (-1.38,1.04) | Low*‡ |
|  | CIS |  |  | **1.18 (0.17,2.19)** | Low*‡ | **1.18 (0.17,2.19)** | Low*‡ |
| GP vs. |  |  |  |  |  |  |  |
|  | GCS |  |  | -0.57 (-1.69,0.56) | Low*‡ | -0.57 (-1.69,0.56) | Low*‡ |
|  | GCG |  |  | -0.74 (-1.87,0.40) | Low*‡ | -0.74 (-1.87,0.40) | Low*‡ |
|  | GCCET |  |  | -0.52 (-1.71,0.67) | Low*‡ | -0.52 (-1.71,0.67) | Low*‡ |
|  | GCA |  |  | -0.05 (-1.03,0.94) | Low*‡ | -0.05 (-1.03,0.94) | Low*‡ |
|  | GC | -0.40(-1.30,0.51) | Low*‡ | -0.21(-1.59,1.17) | Low*‡ | -0.34 (-1.10,0.41) | Low*‡ |
|  | GF |  |  | **4.62 (1.99,7.25)** | Low*‡ | **4.62 (1.99,7.25)** | Low*‡ |
|  | FAP |  |  | 0.53 (-0.49,1.54) | Low*‡ | 0.53 (-0.49,1.54) | Low*‡ |
|  | DC |  |  | 0.52 (-0.49,1.53) | Low*‡ | 0.52 (-0.49,1.53) | Low*‡ |
|  | CP |  |  | -0.01 (-1.25,1.23) | Low*‡ | -0.01 (-1.25,1.23) | Low*‡ |
|  | CIS |  |  | **1.34 (0.30,2.38)** | Low*‡ | **1.34 (0.30,2.38)** | Low*‡ |
| GCS vs. |  |  |  |  |  |  |  |
|  | GCG |  |  | -0.17 (-1.36,1.02) | Low*‡ | -0.17 (-1.36,1.02) | Low*‡ |
|  | GCCET |  |  | 0.05 (-1.20,1.29) | Low*‡ | 0.05 (-1.20,1.29) | Low*‡ |
|  | GCA |  |  | 0.52 (-0.53,1.57) | Moderate‡ | 0.52 (-0.53,1.57) | Moderate‡ |
|  | GC | 0.22 (-0.61,1.06) | Moderate‡ | NA | NA | 0.22 (-0.61,1.06) | Moderate‡ |
|  | GF |  |  | **5.19 (2.51,7.87)** | Low*‡ | **5.19 (2.51,7.87)** | Low*‡ |
|  | FAP |  |  | 1.09 (-0.05,2.24) | Low*‡ | 1.09 (-0.05,2.24) | Low*‡ |
|  | DC |  |  | 1.09 (-0.05,2.23) | Low*‡ | 1.09 (-0.05,2.23) | Low*‡ |
|  | CP |  |  | 0.55 (-0.79,1.90) | Low*‡ | 0.55 (-0.79,1.90) | Low*‡ |
|  | CIS |  |  | **1.90 (0.73,3.07)** | Low*‡ | **1.90 (0.73,3.07)** | Low*‡ |
| GCG vs. |  |  |  |  |  |  |  |
|  | GCCET |  |  | 0.22 (-1.03,1.47) | Low*‡ | 0.22 (-1.03,1.47) | Low*‡ |
|  | GCA |  |  | 0.69 (-0.37,1.74) | Low*‡ | 0.69 (-0.37,1.74) | Low*‡ |
|  | GC | 0.39 (-0.45,1.24) | Low*‡ | NA | NA | 0.39 (-0.45,1.24) | Low*‡ |
|  | GF |  |  | **5.36 (2.67,8.04)** | Low*‡ | **5.36 (2.67,8.04)** | Low*‡ |
|  | FAP |  |  | **1.26 (0.11,2.42)** | Low*‡ | **1.26 (0.11,2.42)** | Low*‡ |
|  | DC |  |  | **1.26 (0.11,2.41)** | Low*‡ | **1.26 (0.11,2.41)** | Low*‡ |
|  | CP |  |  | 0.72 (-0.63,2.08) | Low*‡ | 0.72 (-0.63,2.08) | Low*‡ |
|  | CIS |  |  | **2.07 (0.90,3.25)** | Low*‡ | **2.07 (0.90,3.25)** | Low*‡ |
| GCCET vs. |  |  |  |  |  |  |  |
|  | GCA |  |  | 0.47 (-0.64,1.59) | Low*‡ | 0.47 (-0.64,1.59) | Low*‡ |
|  | GC | 0.18 (-0.74,1.10) | Low*‡ | NA | NA | 0.18 (-0.74,1.10) | Low*‡ |
|  | GF |  |  | **5.14 (2.43,7.85)** | Low*‡ | **5.14 (2.43,7.85)** | Low*‡ |
|  | FAP |  |  | 1.05 (-0.16,2.25) | Low*‡ | 1.05 (-0.16,2.25) | Low*‡ |
|  | DC |  |  | 1.04 (-0.16,2.24) | Low*‡ | 1.04 (-0.16,2.24) | Low*‡ |
|  | CP |  |  | 0.51 (-0.89,1.91) | Low*‡ | 0.51 (-0.89,1.91) | Low*‡ |
|  | CIS |  |  | **1.86 (0.63,3.09)** | Low*‡ | **1.86 (0.63,3.09)** | Low*‡ |
| GCA vs. |  |  |  |  |  |  |  |
|  | GC | -0.30 (-0.93,0.33) | Moderate‡ | NA | NA | -0.30 (-0.93,0.33) | Moderate‡ |
|  | GF |  |  | **4.67 (2.04,7.29)** | Low*‡ | **4.67 (2.04,7.29)** | Low*‡ |
|  | FAP |  |  | 0.57 (-0.43,1.58) | Low*‡ | 0.57 (-0.43,1.58) | Low*‡ |
|  | DC |  |  | 0.57 (-0.43,1.57) | Low*‡ | 0.57 (-0.43,1.57) | Low*‡ |
|  | CP |  |  | 0.04 (-1.19,1.26) | Low*‡ | 0.04 (-1.19,1.26) | Low*‡ |
|  | CIS |  |  | **1.38 (0.35,2.42)** | Low*‡ | **1.38 (0.35,2.42)** | Low*‡ |
| GC vs. |  |  |  |  |  |  |  |
|  | GF |  |  | **4.96 (2.42,7.51)** | Low*‡ | **4.96 (2.42,7.51)** | Low*‡ |
|  | FAP |  |  | **0.87 (0.09,1.65)** | Low*‡ | **0.87 (0.09,1.65)** | Low*‡ |
|  | DC |  |  | **0.86 (0.09,1.64)** | Low*‡ | **0.86 (0.09,1.64)** | Low*‡ |
|  | CP |  |  | 0.33 (-0.72,1.39) | Low*‡ | 0.33 (-0.72,1.39) | Low*‡ |
|  | CIS |  |  | **1.68 (0.86,2.50)** | Low*‡ | **1.68 (0.86,2.50)** | Low*‡ |
| GF vs. |  |  |  |  |  |  |  |
|  | FAP |  |  | **-4.09 (-6.67,-1.52)** | Low*‡ | **-4.09 (-6.67,-1.52)** | Low*‡ |
|  | DC |  |  | **-4.10 (-6.67,-1.53)** | Low*‡ | **-4.10 (-6.67,-1.53)** | Low*‡ |
|  | CP |  |  | **-4.63 (-7.30,-1.96)** | Low*‡ | **-4.63 (-7.30,-1.96)** | Low*‡ |
|  | CIS |  |  | **-3.28 (-5.87,-0.70)** | Low*‡ | **-3.28 (-5.87,-0.70)** | Low*‡ |
| FAP vs. |  |  |  |  |  |  |  |
|  | DC |  |  | -0.01 (-0.87,0.85) | Low*‡ | -0.01 (-0.87,0.85) | Low*‡ |
|  | CP |  |  | -0.54 (-1.66,0.58) | Low*‡ | -0.54 (-1.66,0.58) | Low*‡ |
|  | CIS |  |  | 0.81 (-0.09,1.71) | Low*‡ | 0.81 (-0.09,1.71) | Low*‡ |
| DC vs. |  |  |  |  |  |  |  |
|  | CP |  |  | -0.53 (-1.65,0.58) | Low*‡ | -0.53 (-1.65,0.58) | Low*‡ |
|  | CIS |  |  | 0.82 (-0.08,1.71) | Low*‡ | 0.82 (-0.08,1.71) | Low*‡ |
| CP vs. |  |  |  |  |  |  |  |
|  | CIS |  |  | **1.35 (0.20,2.50)** | Low*‡ | **1.35 (0.20,2.50)** | Low*‡ |

Abbreviations: CIs: confidence intervals; LogOR: logarithm hazard ratio; NA: not available.

Abbreviations of intervention are showed in Table 1.

Bold means statistic difference (p<0.05).

*: Study limitation; †: Large-scale effect; ‡: Imprecision; #: Incoherence.
